# Supplementary material for: Polyketide synthase gene clusters in the ericoid mycorrhizal fungus Oidiodendron maius: genome-wide prediction, expression profiling, and functional characterization of knockout mutants
Source: Front Plant Sci. 2026 Sep 16;17:1924430. doi: 10.3389/fpls.2026.1924430 (PMC13623903; doi:10.3389/fpls.2026.1924430)
Supplement: Supplementary file 1 [file SupplementaryFile1.zip › Supplementary Materials/Supplementary Folder 1.zip!/Oidma1_AssemblyScaffolds_Repeatmasked/knownclusterblast/cluster2/jgi.p_Oidma1_36638_mibig_hits.html]

| MIBiG Protein | Description | MIBiG Cluster | MiBiG Product | % ID | % Coverage | BLAST Score | E-value |
| --- | --- | --- | --- | --- | --- | --- | --- |
| CCE67070.1 | polyketide\_synthase | BGC0001242 | Fusarubin\_biosynthetic\_gene\_cluster | 61 | 100.618647813 | 2802 | 0.0 |
| AAS90093.1 | PksA | BGC0000006 | Aflatoxin\_biosynthetic\_gene\_cluster | 44 | 79.6730004419 | 1459 | 0.0 |
| ACH72912.1 | AflC | BGC0000011 | Aflatoxin/sterigmatocystin\_biosynthetic\_gene\_cluster | 43 | 86.5665046399 | 1459 | 0.0 |
| BAE71314.1 | polyketide\_synthase | BGC0000004 | Aflatoxin\_biosynthetic\_gene\_cluster | 44 | 79.7171895714 | 1456 | 0.0 |
| CBF74114.1 | Conidial\_yellow\_pigment\_biosynthesis\_polyketide\_synthase\_(PKS)(EC\_2.3.1.-)\_[Source:UniProtKB/Swiss-Prot;Acc:Q03149] | BGC0000107 | Naphthopyrone\_biosynthetic\_gene\_cluster | 43 | 83.3406981882 | 1455 | 0.0 |
| AAS89999.1 | PksA | BGC0000007 | Aflatoxin\_biosynthetic\_gene\_cluster | 44 | 81.9266460451 | 1454 | 0.0 |
| AAS90022.1 | PksA | BGC0000008 | Aflatoxin\_biosynthetic\_gene\_cluster | 44 | 79.7171895714 | 1454 | 0.0 |
| AAS90047.1 | PksA | BGC0000009 | Aflatoxin\_biosynthetic\_gene\_cluster | 44 | 81.7057003977 | 1437 | 0.0 |
| AAT69682.1 | polyketide\_synthase | BGC0001247 | Cercosporin\_biosynthetic\_gene\_cluster | 41 | 84.1361025188 | 1374 | 0.0 |
| AAC49191.1 | putative\_polyketide\_synthase | BGC0000152 | Sterigmatocystin\_biosynthetic\_gene\_cluster | 42 | 85.9478568272 | 1364 | 0.0 |
| ABU63483.1 | polyketide\_synthase | BGC0001259 | Elsinochrome\_A\_biosynthetic\_gene\_cluster | 39 | 84.4454264251 | 1305 | 0.0 |
| BAD22832.1 | polyketide\_synthase | BGC0001265 | Melanin\_biosynthetic\_gene\_cluster | 38 | 86.3455589925 | 1278 | 0.0 |
| AAD38786.1 | polyketide\_synthase | BGC0001257 | 1,3,6,8-tetrahydroxynaphthalene\_biosynthetic\_gene\_cluster | 40 | 81.7498895272 | 1262 | 0.0 |
| AAN59953.1 | polyketide\_synthase\_1 | BGC0001258 | 1,8-dihydroxynaphthalene\_biosynthetic\_gene\_cluster | 39 | 82.0592134335 | 1258 | 0.0 |
| CCT67991.1 | bikaverin\_cluster-polyketide\_synthase | BGC0000030 | Bikaverin\_biosynthetic\_gene\_cluster | 40 | 81.3079982324 | 1253 | 0.0 |
| CCH26295.1 | putative\_polyketide\_synthase | BGC0001158\_c6 | Bikaverin\_biosynthetic\_gene\_cluster | 40 | 78.656650464 | 1208 | 0.0 |
| EAU38791.1 | hypothetical\_protein | BGC0000161 | Terrein\_biosynthetic\_gene\_cluster | 38 | 81.6615112682 | 1204 | 0.0 |
| AAZ95017.1 | polyketide\_synthase | BGC0000048 | Dothistromin\_biosynthetic\_gene\_cluster | 47 | 59.3018117543 | 1178 | 0.0 |
| ADM79459.1 | PKS16\_protein | BGC0001266 | Grayanic\_acid\_biosynthetic\_gene\_cluster | 38 | 82.6778612461 | 1154 | 0.0 |
| EAL89339.1 | polyketide\_synthase,\_putative | BGC0001403 | Trypacidin\_biosynthetic\_gene\_cluster | 39 | 77.2425983208 | 1132 | 0.0 |
| AGO59040.1 | PtaA | BGC0000121 | Pestheic\_acid\_biosynthetic\_gene\_cluster | 38 | 75.5634114008 | 1105 | 0.0 |
| CBF90097.1 | hypothetical\_protein\_similar\_to\_polyketide\_synthase\_(Broad) | BGC0001271 | Emericellin\_biosynthetic\_gene\_cluster | 37 | 79.0101634998 | 1091 | 0.0 |
| AAS92537.1 | PKS1 | BGC0001044 | Sirodesmin\_biosynthetic\_gene\_cluster | 46 | 56.2969509501 | 1070 | 3.2e-312 |
| AEN83889.1 | AdaA | BGC0000156 | TAN-1612\_biosynthetic\_gene\_cluster | 36 | 78.0821917808 | 1040 | 3.6e-303 |
| EGD99348.1 | polyketide\_synthase | BGC0001144 | Neosartoricin\_biosynthetic\_gene\_cluster | 36 | 79.1869200177 | 1037 | 1.8e-302 |
| EAL84397.1 | polyketide\_synthase,\_putative | BGC0001118 | Endocrocin\_biosynthetic\_gene\_cluster | 37 | 70.6142288997 | 1010 | 2.3e-294 |
| CBF70387.1 | polyketide\_synthase,\_putative\_(JCVI) | BGC0000684 | Asperthecin\_biosynthetic\_gene\_cluster | 36 | 72.7794962439 | 1009 | 6.7e-294 |
| ADI24926.1 | VrtA | BGC0000168 | Viridicatumtoxin\_biosynthetic\_gene\_cluster | 37 | 74.0167918692 | 998 | 9.1e-291 |
| EED53479.1 | polyketide\_synthase,\_putative | BGC0001304 | Aflavarin\_biosynthetic\_gene\_cluster | 34 | 77.3751657092 | 990 | 2.5e-288 |
| AKN45693.1 | polyketide\_synthase | BGC0001284 | Alternariol\_biosynthetic\_gene\_cluster | 36 | 70.7026071586 | 944 | 2.7e-274 |
| ADI24953.1 | GsfA | BGC0000070 | Griseofulvin\_biosynthetic\_gene\_cluster | 34 | 72.3376049492 | 908 | 1.2e-263 |
| CBF79143.1 | polyketide\_synthase,\_putative\_(JCVI) | BGC0000013 | Alternariol\_biosynthetic\_gene\_cluster | 33 | 76.3146266019 | 853 | 6.2e-247 |
| ACD39753.1 | non-reducing\_polyketide\_synthase | BGC0000076 | Hypothemycin\_biosynthetic\_gene\_cluster | 31 | 78.656650464 | 786 | 7.1e-227 |
| ACD39762.1 | non-reducing\_polyketide\_synthase | BGC0000077 | Hypothemycin\_biosynthetic\_gene\_cluster | 31 | 78.656650464 | 785 | 2.1e-226 |
| ABB90282.1 | polyketide\_synthase | BGC0001057 | Zearalenone\_biosynthetic\_gene\_cluster | 32 | 72.9562527618 | 781 | 2.3e-225 |
| AHV78253.1 | ResS2 | BGC0001246 | Trans-resorcylide\_biosynthetic\_gene\_cluster | 30 | 82.5894829872 | 771 | 3.1e-222 |
| AGC95321.1 | CurS2 | BGC0000045 | Dehydrocurvularin\_biosynthetic\_gene\_cluster | 32 | 75.3424657534 | 770 | 4e-222 |
| EAA59563.1 | hypothetical\_protein | BGC0000057 | F9775\_biosynthetic\_gene\_cluster | 30 | 82.5894829872 | 769 | 1.2e-221 |
| AHV78247.1 | LasS2 | BGC0001245 | Lasiodiplodin\_biosynthetic\_gene\_cluster | 30 | 79.6288113124 | 736 | 8.4e-212 |
| ACD39770.1 | non-reducing\_polyketide\_synthase | BGC0000134 | Radicicol\_biosynthetic\_gene\_cluster | 30 | 73.442333186 | 708 | 1.9e-203 |
| AEU11005.1 | NpnA | BGC0001029 | Nostophycin\_biosynthetic\_gene\_cluster | 35 | 39.5050817499 | 519 | 2.8e-146 |
| XP\_011392701.1 | hypothetical\_protein | BGC0001281 | Ustilagic\_acid\_biosynthetic\_gene\_cluster | 29 | 54.0433053469 | 510 | 1.3e-143 |
| AAT70105.1 | CurJ | BGC0001165 | Curacin\_biosynthetic\_gene\_cluster | 29 | 55.1922227132 | 503 | 1.2e-141 |
| AEE88280.1 | CurJ | BGC0000976 | Curacin\_biosynthetic\_gene\_cluster | 29 | 55.1922227132 | 503 | 1.2e-141 |
| AFV96138.1 | polyketide\_synthase | BGC0001064 | Cylindrocyclophane\_biosynthetic\_gene\_cluster | 35 | 40.3446752099 | 501 | 6e-141 |
| AMB48442.1 | polyketide\_synthase | BGC0001357 | Carbamidocyclophane\_biosynthetic\_gene\_cluster | 34 | 40.1237295625 | 500 | 7.8e-141 |
| AEU11006.1 | NpnB | BGC0001029 | Nostophycin\_biosynthetic\_gene\_cluster | 34 | 39.8585947857 | 500 | 1.3e-140 |
| AAT70104.1 | CurI | BGC0001165 | Curacin\_biosynthetic\_gene\_cluster | 33 | 40.4772425983 | 498 | 3e-140 |
| AEE88281.1 | CurI | BGC0000976 | Curacin\_biosynthetic\_gene\_cluster | 33 | 40.4772425983 | 498 | 3e-140 |
| AAS98783.1 | JamL | BGC0001001 | Jamaicamide\_biosynthetic\_gene\_cluster | 30 | 51.7896597437 | 498 | 5.1e-140 |
| AIW82279.1 | PuwB | BGC0001125 | Puwainaphycins\_biosynthetic\_gene\_cluster | 30 | 53.1595227574 | 495 | 2.5e-139 |
| ABX60162.1 | polyketide\_synthase | BGC0000978 | Cylindrospermopsin\_biosynthetic\_gene\_cluster | 34 | 39.6376491383 | 495 | 4.3e-139 |
| AGN71604.1 | conidial\_yellow\_pigment\_biosynthesis\_polyketide\_synthase | BGC0000027 | Azaphilone\_biosynthetic\_gene\_cluster | 27 | 82.015024304 | 493 | 1.2e-138 |
| ADF88280.1 | polyketide\_synthase | BGC0000981 | Cylindrospermopsin\_biosynthetic\_gene\_cluster | 34 | 39.7702165267 | 493 | 1.6e-138 |
| ADY00130.1 | polyketide\_synthase | BGC0000104 | Mycophenolic\_acid\_biosynthetic\_gene\_cluster | 28 | 70.083959346 | 489 | 2.3e-137 |
| ACR33078.1 | polyketide\_synthase | BGC0000017 | Anatoxin\_biosynthetic\_gene\_cluster | 32 | 44.7635881573 | 489 | 2.3e-137 |
| AAT70107.1 | CurL | BGC0001165 | Curacin\_biosynthetic\_gene\_cluster | 34 | 40.7865665046 | 488 | 5.2e-137 |
| AEE88278.1 | CurL | BGC0000976 | Curacin\_biosynthetic\_gene\_cluster | 34 | 40.7865665046 | 488 | 5.2e-137 |
| CBD77748.1 | polyketide\_synthase | BGC0000974 | Crocacin\_biosynthetic\_gene\_cluster | 30 | 55.2364118427 | 487 | 6.8e-137 |
| CAQ18828.1 | polyketide\_synthase | BGC0000954 | Ajudazol\_biosynthetic\_gene\_cluster | 35 | 40.256296951 | 487 | 1.2e-136 |
| ABX60163.1 | polyketide\_synthase | BGC0000978 | Cylindrospermopsin\_biosynthetic\_gene\_cluster | 32 | 41.8912947415 | 485 | 4.4e-136 |
| CAQ18829.1 | polyketide\_synthase | BGC0000954 | Ajudazol\_biosynthetic\_gene\_cluster | 32 | 45.1612903226 | 483 | 9.9e-136 |
| ADF88277.1 | polyketide\_synthase | BGC0000981 | Cylindrospermopsin\_biosynthetic\_gene\_cluster | 31 | 44.6752098984 | 483 | 1.3e-135 |
| AAS98777.1 | JamE | BGC0001001 | Jamaicamide\_biosynthetic\_gene\_cluster | 34 | 41.007512152 | 482 | 2.2e-135 |
| CAQ18834.1 | polyketide\_synthase | BGC0000954 | Ajudazol\_biosynthetic\_gene\_cluster | 35 | 39.9469730446 | 482 | 3.7e-135 |
| AHB82053.1 | polyketide\_synthase | BGC0001019 | Microsclerodermins\_biosynthetic\_gene\_cluster | 35 | 39.6376491383 | 481 | 6.4e-135 |
| ABM21570.1 | CrpB | BGC0000975 | Cryptophycin\_biosynthetic\_gene\_cluster | 29 | 55.1922227132 | 480 | 8.3e-135 |
| ADN13832.1 | Beta-ketoacyl\_synthase | BGC0001164 | 1-heptadecene\_biosynthetic\_gene\_cluster | 33 | 41.2284577994 | 480 | 8.3e-135 |
| ACR33077.1 | polyketide\_synthase | BGC0000017 | Anatoxin\_biosynthetic\_gene\_cluster | 31 | 51.1268228016 | 478 | 5.4e-134 |
| AEE88289.1 | CurA | BGC0000976 | Curacin\_biosynthetic\_gene\_cluster | 32 | 42.0238621299 | 477 | 1.2e-133 |
| AAT70096.1 | CurA | BGC0001165 | Curacin\_biosynthetic\_gene\_cluster | 32 | 42.0238621299 | 477 | 1.2e-133 |
| BAV19379.1 | polyketide\_synthase | BGC0001390 | LL-Z1272beta\_biosynthetic\_gene\_cluster | 30 | 56.8714096332 | 477 | 1.2e-133 |
| ADF88276.1 | polyketide\_synthase | BGC0000981 | Cylindrospermopsin\_biosynthetic\_gene\_cluster | 31 | 44.2333186036 | 476 | 2.1e-133 |
| AAW03329.1 | CtaF | BGC0000982 | Cystothiazole\_A\_biosynthetic\_gene\_cluster | 32 | 41.095890411 | 475 | 3.5e-133 |
| AAF19813.1 | MtaE | BGC0001024 | Myxothiazol\_biosynthetic\_gene\_cluster | 35 | 40.167918692 | 475 | 3.5e-133 |
| AAF26922.1 | polyketide\_synthase | BGC0000988 | Epothilone\_biosynthetic\_gene\_cluster | 30 | 53.026955369 | 474 | 6e-133 |
| AGC45619.1 | polyketide\_synthase | BGC0001394 | Phenalamide\_biosynthetic\_gene\_cluster | 31 | 51.347768449 | 474 | 7.8e-133 |
| ADB12492.1 | EpoE | BGC0000990 | Epothilone\_biosynthetic\_gene\_cluster | 30 | 52.7176314627 | 473 | 1.3e-132 |
| AEE88277.1 | CurM | BGC0000976 | Curacin\_biosynthetic\_gene\_cluster | 32 | 42.6425099426 | 473 | 1.3e-132 |
| ACV42478.1 | polyketide\_synthase | BGC0000043 | Curacin\_A\_biosynthetic\_gene\_cluster | 32 | 42.6425099426 | 473 | 1.3e-132 |
| AAT70108.1 | CurM | BGC0001165 | Curacin\_biosynthetic\_gene\_cluster | 32 | 42.6425099426 | 473 | 1.3e-132 |
| CAD89777.1 | MelF\_protein | BGC0001009 | Melithiazol\_biosynthetic\_gene\_cluster | 33 | 39.8144056562 | 472 | 2.3e-132 |
| AAF62884.1 | EpoE | BGC0000991 | Epothilone\_biosynthetic\_gene\_cluster | 30 | 52.7176314627 | 472 | 3.9e-132 |
| ACB46196.1 | polyketide\_synthase | BGC0000989 | Epothilone\_biosynthetic\_gene\_cluster | 30 | 52.9827662395 | 472 | 3.9e-132 |
| AAF62883.1 | epoD | BGC0000991 | Epothilone\_biosynthetic\_gene\_cluster | 34 | 40.6539991162 | 471 | 6.6e-132 |
| ADZ24998.1 | polyketide\_synthase | BGC0000380 | Leupyrrin\_biosynthetic\_gene\_cluster | 33 | 40.4772425983 | 470 | 8.6e-132 |
| AAK57189.1 | MxaE | BGC0001022 | Myxalamid\_biosynthetic\_gene\_cluster | 31 | 45.1612903226 | 470 | 8.6e-132 |
| AAO62584.1 | polyketide\_synthase\_type\_1 | BGC0001016 | Microcystin\_biosynthetic\_gene\_cluster | 29 | 56.2969509501 | 470 | 1.1e-131 |
| ADB12491.1 | EpoD | BGC0000990 | Epothilone\_biosynthetic\_gene\_cluster | 34 | 40.6539991162 | 470 | 1.5e-131 |
| ABX60152.1 | polyketide\_synthase | BGC0000978 | Cylindrospermopsin\_biosynthetic\_gene\_cluster | 31 | 44.2333186036 | 469 | 2.5e-131 |
| CAQ34920.1 | polyketide\_synthase | BGC0000986 | Dkxanthene\_biosynthetic\_gene\_cluster | 30 | 50.8174988953 | 468 | 4.3e-131 |
| AAF26921.1 | polyketide\_synthase | BGC0000988 | Epothilone\_biosynthetic\_gene\_cluster | 33 | 40.6539991162 | 466 | 2.1e-130 |
| CAQ18833.1 | polyketide\_synthase | BGC0000954 | Ajudazol\_biosynthetic\_gene\_cluster | 33 | 40.256296951 | 466 | 2.1e-130 |
| AFU82617.1 | polyketide\_synthase | BGC0000998 | Guadinomine\_biosynthetic\_gene\_cluster | 33 | 39.9911621741 | 465 | 2.8e-130 |
| AHN85651.1 | Phn2 | BGC0000122 | Phenylnannolone\_A\_biosynthetic\_gene\_cluster | 33 | 41.095890411 | 463 | 1.4e-129 |
| AAK19883.1 | soraphen\_polyketide\_synthase\_A | BGC0000147 | Soraphen\_biosynthetic\_gene\_cluster | 33 | 40.9633230225 | 463 | 1.4e-129 |
| AHH34186.1 | polyketide\_synthase | BGC0001161 | 1-heptadecene\_biosynthetic\_gene\_cluster | 31 | 43.1285903668 | 463 | 1.8e-129 |
| ACB46195.1 | polyketide\_synthase | BGC0000989 | Epothilone\_biosynthetic\_gene\_cluster | 33 | 40.6539991162 | 462 | 3.1e-129 |
| CAD19093.1 | StiJ\_protein | BGC0000153 | Stigmatellin\_biosynthetic\_gene\_cluster | 33 | 41.8912947415 | 462 | 4e-129 |
| AAF19810.1 | MtaB | BGC0001024 | Myxothiazol\_biosynthetic\_gene\_cluster | 32 | 45.38223597 | 461 | 5.2e-129 |
| CAQ18830.1 | polyketide\_synthase | BGC0000954 | Ajudazol\_biosynthetic\_gene\_cluster | 34 | 44.1891294741 | 461 | 5.2e-129 |
| AAW03328.1 | CtaE | BGC0000982 | Cystothiazole\_A\_biosynthetic\_gene\_cluster | 34 | 40.5656208573 | 461 | 6.8e-129 |
| AEE88282.1 | CurH | BGC0000976 | Curacin\_biosynthetic\_gene\_cluster | 33 | 39.9911621741 | 460 | 8.9e-129 |
| AAT70103.1 | CurH | BGC0001165 | Curacin\_biosynthetic\_gene\_cluster | 33 | 39.9911621741 | 460 | 8.9e-129 |
| ACR33079.1 | polyketide\_synthase | BGC0000017 | Anatoxin\_biosynthetic\_gene\_cluster | 33 | 39.8585947857 | 460 | 8.9e-129 |
| AID65222.1 | putative\_aspartate\_racemase | BGC0000335 | Cystomanamides\_biosynthetic\_gene\_cluster | 33 | 41.5819708352 | 460 | 1.2e-128 |
| CAQ18835.1 | polyketide\_synthase | BGC0000954 | Ajudazol\_biosynthetic\_gene\_cluster | 35 | 40.0795404331 | 458 | 3.4e-128 |
| AAK57187.1 | MxaC | BGC0001022 | Myxalamid\_biosynthetic\_gene\_cluster | 34 | 42.4215642952 | 458 | 3.4e-128 |
| CAD19088.1 | StiD\_protein | BGC0000153 | Stigmatellin\_biosynthetic\_gene\_cluster | 33 | 40.2121078215 | 457 | 9.9e-128 |
| CAO98850.1 | polyketide\_synthase\_AufG | BGC0000023 | Aurafuron\_biosynthetic\_gene\_cluster | 34 | 40.1237295625 | 456 | 2.2e-127 |
| AAF26919.1 | polyketide\_synthase | BGC0000988 | Epothilone\_biosynthetic\_gene\_cluster | 34 | 40.167918692 | 450 | 1.6e-125 |
| AFU82615.1 | polyketide\_synthase | BGC0000998 | Guadinomine\_biosynthetic\_gene\_cluster | 34 | 39.5492708794 | 449 | 2.1e-125 |
| AHA12079.1 | polyketide\_synthase\_type\_1 | BGC0001172 | Chlorizidine\_A\_biosynthetic\_gene\_cluster | 34 | 39.9027839152 | 448 | 3.5e-125 |
| CAD89776.1 | MelE\_protein | BGC0001009 | Melithiazol\_biosynthetic\_gene\_cluster | 32 | 41.5377817057 | 448 | 4.6e-125 |
| CDM36726.1 | Beta-ketoacyl\_synthase | BGC0001360 | Mycophenolic\_acid\_biosynthetic\_gene\_cluster | 28 | 60.3181617322 | 448 | 6e-125 |
| AGC45624.1 | polyketide\_synthase | BGC0001394 | Phenalamide\_biosynthetic\_gene\_cluster | 30 | 52.8060097216 | 446 | 1.7e-124 |
| AAW03325.1 | CtaB | BGC0000982 | Cystothiazole\_A\_biosynthetic\_gene\_cluster | 34 | 40.167918692 | 446 | 2.3e-124 |
| AAU04878.1 | polyketide\_synthase | BGC0000365 | Glycopeptidolipid\_biosynthetic\_gene\_cluster | 31 | 43.3053468847 | 445 | 3e-124 |
| AAS98781.1 | JamJ | BGC0001001 | Jamaicamide\_biosynthetic\_gene\_cluster | 27 | 54.3526292532 | 445 | 3e-124 |
| AIT55263.1 | GulF | BGC0000072 | Gulmirecin\_biosynthetic\_gene\_cluster | 33 | 40.4772425983 | 445 | 3.9e-124 |
| AAF62880.1 | EpoA | BGC0000991 | Epothilone\_biosynthetic\_gene\_cluster | 33 | 40.167918692 | 444 | 6.6e-124 |
| ADB12488.1 | EpoA | BGC0000990 | Epothilone\_biosynthetic\_gene\_cluster | 33 | 40.167918692 | 444 | 8.7e-124 |
| CAL58686.1 | polyketide\_synthase | BGC0000149 | Spirangien\_biosynthetic\_gene\_cluster | 32 | 40.3888643394 | 443 | 1.1e-123 |
| ACB46192.1 | polyketide\_synthase | BGC0000989 | Epothilone\_biosynthetic\_gene\_cluster | 34 | 40.167918692 | 443 | 1.1e-123 |
| AHA12078.1 | polyketide\_synthase\_type\_1 | BGC0001172 | Chlorizidine\_A\_biosynthetic\_gene\_cluster | 33 | 40.167918692 | 443 | 1.5e-123 |
| CAO98847.1 | polyketide\_synthase\_AufC | BGC0000023 | Aurafuron\_biosynthetic\_gene\_cluster | 33 | 41.6703490941 | 443 | 1.5e-123 |
| CCE88379.1 | polyketide\_synthase | BGC0001034 | Pellasoren\_biosynthetic\_gene\_cluster | 30 | 51.4361467079 | 443 | 1.5e-123 |
| CCE88378.1 | polyketide\_synthase | BGC0001034 | Pellasoren\_biosynthetic\_gene\_cluster | 31 | 43.7472381794 | 441 | 7.3e-123 |
| CAL58685.1 | polyketide\_synthase | BGC0000149 | Spirangien\_biosynthetic\_gene\_cluster | 32 | 40.5656208573 | 441 | 7.3e-123 |
| AAQ82561.1 | FscA | BGC0000061 | FR-008\_biosynthetic\_gene\_cluster | 33 | 42.5983208131 | 440 | 1.6e-122 |
| ALA62323.1 | methylphloroacetophenone\_synthase | BGC0001309 | Usnic\_acid\_biosynthetic\_gene\_cluster | 29 | 51.5245249669 | 440 | 1.6e-122 |
| ABX60161.1 | mixed\_NRPS/PKS | BGC0000978 | Cylindrospermopsin\_biosynthetic\_gene\_cluster | 30 | 46.1776403005 | 438 | 4.8e-122 |
| ADZ24996.1 | polyketide\_synthase | BGC0000380 | Leupyrrin\_biosynthetic\_gene\_cluster | 30 | 52.1431727795 | 438 | 4.8e-122 |
| BAJ16470.1 | polyketide\_synthase | BGC0000058 | FD-891\_biosynthetic\_gene\_cluster | 31 | 43.9239946973 | 438 | 4.8e-122 |
| BAH02271.1 | polyketide\_synthase | BGC0000126 | Pladienolide\_biosynthetic\_gene\_cluster | 32 | 40.6539991162 | 438 | 6.2e-122 |
| BAD08359.1 | polyketide\_synthase\_modules\_5-6 | BGC0000167 | Vicenistatin\_biosynthetic\_gene\_cluster | 32 | 39.7260273973 | 437 | 1.1e-121 |
| CBD77738.1 | polyketide\_synthase | BGC0000974 | Crocacin\_biosynthetic\_gene\_cluster | 33 | 40.2121078215 | 436 | 1.4e-121 |
| CAC20921.1 | PimS2\_protein | BGC0000125 | Pimaricin\_biosynthetic\_gene\_cluster | 31 | 45.293857711 | 436 | 1.8e-121 |
| CBF80428.1 | polyketide\_synthase,\_putative\_(JCVI) | BGC0000680 | Austinol/dehydroaustinol,\_locus\_1\_biosynthetic\_gene\_cluster | 27 | 68.8024745913 | 436 | 1.8e-121 |
| AAK57186.1 | MxaB2 | BGC0001022 | Myxalamid\_biosynthetic\_gene\_cluster | 34 | 36.809544852 | 436 | 2.4e-121 |
| ACN69988.1 | polyketide\_synthase | BGC0000079 | Indanomycin\_biosynthetic\_gene\_cluster | 32 | 41.095890411 | 436 | 2.4e-121 |
| ADZ24997.1 | polyketide\_synthase | BGC0000380 | Leupyrrin\_biosynthetic\_gene\_cluster | 29 | 51.6570923553 | 435 | 3.1e-121 |
| BAD08373.1 | polyketide\_synthase\_modules\_1-3 | BGC0000167 | Vicenistatin\_biosynthetic\_gene\_cluster | 28 | 52.6734423332 | 435 | 4e-121 |
| CCE88376.1 | polyketide\_synthase | BGC0001034 | Pellasoren\_biosynthetic\_gene\_cluster | 33 | 40.167918692 | 435 | 4e-121 |
| CAD19090.1 | StiF\_protein | BGC0000153 | Stigmatellin\_biosynthetic\_gene\_cluster | 32 | 39.8144056562 | 434 | 6.9e-121 |
| ADF88279.1 | mixed\_NRPS/PKS | BGC0000981 | Cylindrospermopsin\_biosynthetic\_gene\_cluster | 30 | 46.1776403005 | 434 | 6.9e-121 |
| BAJ16468.1 | polyketide\_synthase | BGC0000058 | FD-891\_biosynthetic\_gene\_cluster | 31 | 43.5704816615 | 434 | 6.9e-121 |
| AAA79984.2 | soraphen\_polyketide\_synthase\_B | BGC0000147 | Soraphen\_biosynthetic\_gene\_cluster | 32 | 39.9027839152 | 434 | 6.9e-121 |
| CCE88380.1 | polyketide\_synthase | BGC0001034 | Pellasoren\_biosynthetic\_gene\_cluster | 30 | 51.2152010605 | 434 | 9e-121 |
| CBD77732.1 | polyketide\_synthase | BGC0000974 | Crocacin\_biosynthetic\_gene\_cluster | 32 | 42.2448077773 | 433 | 1.2e-120 |
| AEZ64503.1 | Herd | BGC0001065 | Herboxidiene\_biosynthetic\_gene\_cluster | 33 | 39.7702165267 | 432 | 3.4e-120 |
| AAZ77693.1 | ChlA1 | BGC0000036 | Chlorothricin\_biosynthetic\_gene\_cluster | 32 | 40.5656208573 | 431 | 4.4e-120 |
| ACY13415.1 | KR\_domain\_protein | BGC0001367 | Haliamide\_biosynthetic\_gene\_cluster | 32 | 39.6818382678 | 431 | 4.4e-120 |
| CAG28678.1 | polyketide\_synthase | BGC0001023 | Myxochromide\_biosynthetic\_gene\_cluster | 30 | 47.2823685373 | 431 | 4.4e-120 |
| AAZ77694.1 | ChlA2 | BGC0000036 | Chlorothricin\_biosynthetic\_gene\_cluster | 30 | 45.2054794521 | 431 | 5.8e-120 |
| ABP55493.1 | thioester\_reductase\_domain | BGC0001006 | Lymphostin\_biosynthetic\_gene\_cluster | 31 | 40.6098099867 | 431 | 5.8e-120 |
| CAD89773.1 | MelB\_protein | BGC0001009 | Melithiazol\_biosynthetic\_gene\_cluster | 33 | 40.5214317278 | 431 | 5.8e-120 |
| CQR60493.1 | Polyketide\_synthase,\_type\_I,\_modules:\_9\_and\_10 | BGC0001287 | Chaxamycin\_A\_/\_chaxamycin\_B\_/\_chaxamycin\_C\_/\_chaxamycin\_D | 33 | 40.0353513036 | 430 | 1.3e-119 |
| AGC24270.1 | PrlP | BGC0001038 | Pyralomicin\_biosynthetic\_gene\_cluster | 33 | 39.9911621741 | 430 | 1.3e-119 |
| CAQ34928.1 | polyketide\_synthase | BGC0000986 | Dkxanthene\_biosynthetic\_gene\_cluster | 32 | 43.9239946973 | 430 | 1.3e-119 |
| AIT55261.1 | GulD | BGC0000072 | Gulmirecin\_biosynthetic\_gene\_cluster | 33 | 40.0795404331 | 430 | 1.3e-119 |
| AFI57007.1 | QmnA3 | BGC0000133 | Quartromicin\_biosynthetic\_gene\_cluster | 33 | 39.8144056562 | 430 | 1.7e-119 |
| AFU82616.1 | polyketide\_synthase | BGC0000998 | Guadinomine\_biosynthetic\_gene\_cluster | 33 | 40.0353513036 | 430 | 1.7e-119 |
| CAD19089.1 | StiE\_protein | BGC0000153 | Stigmatellin\_biosynthetic\_gene\_cluster | 32 | 39.6818382678 | 428 | 3.8e-119 |
| AAO62585.1 | peptide\_sythetase\_polyketide\_synthase\_fusion\_protein | BGC0001016 | Microcystin\_biosynthetic\_gene\_cluster | 31 | 44.1007512152 | 428 | 4.9e-119 |
| BAR73021.1 | putative\_PKS\_(KS-AT-KR-ACP) | BGC0001194 | Hitachimycin\_biosynthetic\_gene\_cluster | 32 | 39.9469730446 | 427 | 8.4e-119 |
| ABJ97437.1 | MerA | BGC0001012 | Meridamycin\_biosynthetic\_gene\_cluster | 31 | 41.1842686699 | 427 | 1.1e-118 |
| ADX66461.1 | ScnS2 | BGC0000108 | Natamycin\_biosynthetic\_gene\_cluster | 30 | 44.8961555457 | 426 | 1.4e-118 |
| AAG23265.1 | polyketide\_synthase\_extender\_module\_2 | BGC0000148 | Spinosad\_biosynthetic\_gene\_cluster | 32 | 40.2121078215 | 426 | 1.4e-118 |
| AEH42490.1 | polyketide\_synthase | BGC0000032 | Calcimycin\_biosynthetic\_gene\_cluster | 31 | 39.7702165267 | 426 | 1.9e-118 |
| CAQ43077.1 | polyketide\_synthase | BGC0000970 | Chondrochloren\_biosynthetic\_gene\_cluster | 27 | 63.6323464428 | 426 | 1.9e-118 |
| AAZ77698.1 | ChlA5 | BGC0000036 | Chlorothricin\_biosynthetic\_gene\_cluster | 32 | 40.7423773752 | 426 | 1.9e-118 |
| ACB46487.1 | polyketide\_synthase | BGC0000082 | Kijanimicin\_biosynthetic\_gene\_cluster | 30 | 43.4379142731 | 426 | 1.9e-118 |
| BAQ21947.1 | putative\_type\_I\_polyketide\_synthase | BGC0001204 | Versipelostatin\_biosynthetic\_gene\_cluster | 31 | 44.6752098984 | 426 | 2.4e-118 |
| AHH34189.1 | polyketide\_synthase | BGC0001162 | 1-heptadecene\_biosynthetic\_gene\_cluster | 30 | 44.0565620857 | 426 | 2.4e-118 |
| CAJ46690.1 | polyketide\_synthase | BGC0000969 | Chondramide\_biosynthetic\_gene\_cluster | 29 | 54.706142289 | 425 | 4.2e-118 |
| ALI92655.1 | CitS\_citrinin\_polyketide\_synthase | BGC0001338 | Citrinin\_biosynthetic\_gene\_cluster | 27 | 61.3345117101 | 425 | 4.2e-118 |
| BAG84248.1 | putative\_polyketide\_synthase | BGC0000257 | Prodigiosin\_biosynthetic\_gene\_cluster | 32 | 41.7587273531 | 425 | 4.2e-118 |
| ADH01663.1 | putative\_polyketide\_synthase\_PKS3 | BGC0000099 | Monascorubrin\_biosynthetic\_gene\_cluster | 28 | 56.0318161732 | 424 | 7.1e-118 |
| AIT55259.1 | GulB | BGC0000072 | Gulmirecin\_biosynthetic\_gene\_cluster | 32 | 40.3888643394 | 424 | 7.1e-118 |
| ACR50791.1 | putative\_polyketide\_synthase | BGC0000163 | Tetronasin\_biosynthetic\_gene\_cluster | 31 | 39.8585947857 | 423 | 1.2e-117 |
| AAZ77697.1 | ChlA4 | BGC0000036 | Chlorothricin\_biosynthetic\_gene\_cluster | 30 | 45.7799381352 | 423 | 1.2e-117 |
| ctg1\_orf20 |  | BGC0001013 | Meridamycin\_biosynthetic\_gene\_cluster | 32 | 41.4935925762 | 423 | 1.2e-117 |
| ABC87509.1 | polyketide\_synthase | BGC0001011 | Meridamycin\_biosynthetic\_gene\_cluster | 32 | 41.4935925762 | 423 | 1.2e-117 |
| AAF00958.1 | McyE | BGC0001017 | Microcystin\_biosynthetic\_gene\_cluster | 32 | 40.0353513036 | 422 | 2.7e-117 |
| AAF71766.1 | NysI | BGC0000115 | Nystatin\_biosynthetic\_gene\_cluster | 30 | 45.1171011931 | 422 | 2.7e-117 |
| BAQ21939.1 | putative\_type\_I\_polyketide\_synthase | BGC0001204 | Versipelostatin\_biosynthetic\_gene\_cluster | 32 | 45.6915598763 | 421 | 6e-117 |
| AAG23264.1 | polyketide\_synthase\_loading\_and\_extender\_module\_1 | BGC0000148 | Spinosad\_biosynthetic\_gene\_cluster | 30 | 46.2660185594 | 420 | 1e-116 |
| EED18001.1 | polyketide\_synthase,\_putative | BGC0000154 | Stipitatic\_acid\_biosynthetic\_gene\_cluster | 28 | 55.8108705259 | 420 | 1.7e-116 |
| AHA38203.1 | GphJ | BGC0000069 | Gephyronic\_acid\_biosynthetic\_gene\_cluster | 32 | 41.3168360583 | 419 | 2.3e-116 |
| ALP32046.1 | CycF | BGC0001293 | Cyclizidine\_biosynthetic\_gene\_cluster | 29 | 47.2381794079 | 419 | 3e-116 |
| AEC13069.1 | fosC | BGC0000060 | Fostriecin\_biosynthetic\_gene\_cluster | 31 | 42.7308882015 | 419 | 3e-116 |
| BAT51066.1 | type\_I\_polyketide\_synthase | BGC0001296 | Streptazone\_E\_biosynthetic\_gene\_cluster | 32 | 40.4330534688 | 419 | 3e-116 |
| CAO98849.1 | polyketide\_synthase\_AufF | BGC0000023 | Aurafuron\_biosynthetic\_gene\_cluster | 33 | 39.5934600088 | 418 | 3.9e-116 |
| AAZ77696.1 | ChlA3 | BGC0000036 | Chlorothricin\_biosynthetic\_gene\_cluster | 30 | 44.2775077331 | 418 | 3.9e-116 |
| AAG23263.1 | polyketide\_synthase\_extender\_modules\_5-7 | BGC0000148 | Spinosad\_biosynthetic\_gene\_cluster | 31 | 41.8912947415 | 418 | 5.1e-116 |
| ABB05103.1 | LipPks2 | BGC0001003 | Lipomycin\_biosynthetic\_gene\_cluster | 29 | 45.9566946531 | 418 | 5.1e-116 |
| ACO94488.1 | polyketide\_synthase\_type\_I | BGC0000097 | ML-449\_biosynthetic\_gene\_cluster | 30 | 46.5753424658 | 418 | 5.1e-116 |
| CAD17792.1 | probable\_non\_ribosomal\_peptide\_synthetase\_protein | BGC0001363 | Ralsolamycin\_biosynthetic\_gene\_cluster | 32 | 39.5934600088 | 418 | 6.6e-116 |
| AAC69329.1 | type\_I\_polyketide\_synthase\_PikAI | BGC0000094 | Methymycin\_/\_pikromycin\_biosynthetic\_gene\_cluster | 32 | 40.6539991162 | 418 | 6.6e-116 |
| CAE46851.1 | Type\_I\_modular\_polyketide\_synthase | BGC0000103 | Mycolactone\_biosynthetic\_gene\_cluster | 32 | 40.9633230225 | 417 | 1.1e-115 |
| CAE46843.1 | Type\_I\_modular\_polyketide\_synthase | BGC0000103 | Mycolactone\_biosynthetic\_gene\_cluster | 32 | 40.9633230225 | 417 | 1.1e-115 |
| AFL48528.1 | laidlomycin\_polyketide\_synthase\_(module\_7\_and\_module\_8) | BGC0000084 | Laidlomycin\_biosynthetic\_gene\_cluster | 31 | 40.6539991162 | 416 | 1.5e-115 |
| AAX98191.1 | polyketide\_synthase\_type\_I | BGC0000052 | ECO-02301\_biosynthetic\_gene\_cluster | 30 | 51.8338488732 | 416 | 1.9e-115 |
| ACO94483.1 | polyketide\_synthase\_type\_I | BGC0000097 | ML-449\_biosynthetic\_gene\_cluster | 30 | 44.9403446752 | 416 | 1.9e-115 |
| ACO94484.1 | polyketide\_synthase\_type\_I | BGC0000097 | ML-449\_biosynthetic\_gene\_cluster | 31 | 39.9027839152 | 416 | 2.5e-115 |
| CBD77734.1 | polyketide\_synthase | BGC0000974 | Crocacin\_biosynthetic\_gene\_cluster | 31 | 40.0353513036 | 415 | 3.3e-115 |
| CCE88381.1 | polyketide\_synthase | BGC0001034 | Pellasoren\_biosynthetic\_gene\_cluster | 33 | 39.5934600088 | 415 | 3.3e-115 |
| CAC22145.1 | putative\_type\_I\_polyketide\_synthase | BGC0000038 | Coelimycin\_biosynthetic\_gene\_cluster | 31 | 41.095890411 | 415 | 4.3e-115 |
| AHH99921.1 | PKS\_I | BGC0000002 | Aculeximycin\_biosynthetic\_gene\_cluster | 32 | 40.5214317278 | 414 | 7.3e-115 |
| ACB37741.1 | putative\_type\_I\_polyketide\_synthase | BGC0000162 | Tetrocarcin\_A\_biosynthetic\_gene\_cluster | 30 | 46.0008837826 | 414 | 9.6e-115 |
| AAG23262.1 | polyketide\_synthase\_extender\_modules\_8-10 | BGC0000148 | Spinosad\_biosynthetic\_gene\_cluster | 32 | 40.3004860804 | 413 | 1.3e-114 |
| AAW03327.1 | CtaD | BGC0000982 | Cystothiazole\_A\_biosynthetic\_gene\_cluster | 26 | 56.6504639859 | 413 | 1.6e-114 |
| ADH04660.1 | TugD | BGC0001342 | Thuggacin\_biosynthetic\_gene\_cluster | 30 | 43.3053468847 | 413 | 2.1e-114 |
| ABO15861.1 | polyketide\_synthase | BGC0000130 | Pyrrolomycin\_biosynthetic\_gene\_cluster | 32 | 41.4052143173 | 412 | 2.8e-114 |
| BAJ16467.1 | polyketide\_synthase | BGC0000058 | FD-891\_biosynthetic\_gene\_cluster | 31 | 43.4821034026 | 412 | 3.6e-114 |
| AIT55262.1 | GulE | BGC0000072 | Gulmirecin\_biosynthetic\_gene\_cluster | 31 | 40.256296951 | 412 | 3.6e-114 |
| AFP87524.1 | type\_I\_polyketide\_synthase | BGC0001159 | Marinopyrrole\_biosynthetic\_gene\_cluster | 32 | 41.7587273531 | 411 | 4.8e-114 |
| AAP42858.1 | NanA4 | BGC0000105 | Nanchangmycin\_biosynthetic\_gene\_cluster | 32 | 41.4935925762 | 411 | 6.2e-114 |
| ABO15860.1 | polyketide\_synthase | BGC0000130 | Pyrrolomycin\_biosynthetic\_gene\_cluster | 31 | 40.5214317278 | 411 | 6.2e-114 |
| AFL48529.1 | laidlomycin\_polyketide\_synthase\_(module\_5\_and\_module\_6) | BGC0000084 | Laidlomycin\_biosynthetic\_gene\_cluster | 31 | 40.5214317278 | 411 | 8.1e-114 |
| AAS98784.1 | JamM | BGC0001001 | Jamaicamide\_biosynthetic\_gene\_cluster | 30 | 42.7308882015 | 410 | 1.1e-113 |
| CAD29794.1 | peptide\_synthetase | BGC0001015 | Microcystin\_biosynthetic\_gene\_cluster | 30 | 41.4052143173 | 410 | 1.4e-113 |
| AAQ90173.1 | polyketide\_synthase\_type\_I | BGC0000128 | Pyoluteorin\_biosynthetic\_gene\_cluster | 32 | 40.2121078215 | 410 | 1.8e-113 |
| BAQ25513.1 | type\_I\_polyketide\_synthase | BGC0001288 | Maklamicin\_biosynthetic\_gene\_cluster | 30 | 44.6752098984 | 409 | 2.4e-113 |
| ABV83221.1 | CppI | BGC0000116 | Nystatin-like\_Pseudonocardia\_polyene\_biosynthetic\_gene\_cluster | 30 | 40.7423773752 | 409 | 3.1e-113 |
| BAH02269.1 | polyketide\_synthase | BGC0000126 | Pladienolide\_biosynthetic\_gene\_cluster | 31 | 40.0795404331 | 409 | 3.1e-113 |
| AAX98185.1 | polyketide\_synthase\_type\_I | BGC0000052 | ECO-02301\_biosynthetic\_gene\_cluster | 32 | 39.7260273973 | 408 | 4e-113 |
| AKD43753.1 | HerB | BGC0001349 | Heronamide\_biosynthetic\_gene\_cluster | 32 | 41.4935925762 | 408 | 4e-113 |
| AAF19812.1 | MtaD | BGC0001024 | Myxothiazol\_biosynthetic\_gene\_cluster | 28 | 47.812638091 | 408 | 4e-113 |
| AAF00957.1 | McyG | BGC0001017 | Microcystin\_biosynthetic\_gene\_cluster | 30 | 42.8192664605 | 408 | 4e-113 |
| ADC79639.1 | TamAIII | BGC0001052 | Tirandamycin\_biosynthetic\_gene\_cluster | 30 | 44.0123729563 | 408 | 5.3e-113 |
| CAD89775.1 | MelD\_protein | BGC0001009 | Melithiazol\_biosynthetic\_gene\_cluster | 28 | 51.7454706142 | 407 | 9e-113 |
| ACB46488.1 | polyketide\_synthase | BGC0000082 | Kijanimicin\_biosynthetic\_gene\_cluster | 30 | 45.6915598763 | 407 | 1.2e-112 |
| AAF26920.1 | polyketide\_synthase | BGC0000988 | Epothilone\_biosynthetic\_gene\_cluster | 29 | 45.9125055236 | 406 | 1.5e-112 |
| AHA38199.1 | GphF | BGC0000069 | Gephyronic\_acid\_biosynthetic\_gene\_cluster | 28 | 53.2037118869 | 406 | 2e-112 |
| BAF02923.1 | type\_I\_polyketide\_synthase | BGC0000073 | Halstoctacosanolide\_biosynthetic\_gene\_cluster | 31 | 39.6818382678 | 406 | 2e-112 |
| ACO94460.1 | polyketide\_synthase\_type\_I | BGC0000029 | BE-14106\_biosynthetic\_gene\_cluster | 32 | 42.5541316836 | 406 | 2.6e-112 |
| AEC13080.1 | fosB | BGC0000060 | Fostriecin\_biosynthetic\_gene\_cluster | 31 | 40.6981882457 | 406 | 2.6e-112 |
| ABI91470.1 | beta-ketoacyl\_synthase | BGC0001094 | Enacyloxin\_biosynthetic\_gene\_cluster | 32 | 41.0517012815 | 406 | 2.6e-112 |
| AAN32979.1 | BarE | BGC0000962 | Barbamide\_biosynthetic\_gene\_cluster | 30 | 41.3610251878 | 405 | 3.4e-112 |
| AAS98787.1 | JamP | BGC0001001 | Jamaicamide\_biosynthetic\_gene\_cluster | 32 | 40.3004860804 | 405 | 3.4e-112 |
| AHF22854.1 | MarL | BGC0000091 | Marineosin\_biosynthetic\_gene\_cluster | 31 | 45.0729120636 | 405 | 4.5e-112 |
| BAF85839.1 | modular\_polyketide\_synthase | BGC0000109 | Nemadectin\_biosynthetic\_gene\_cluster | 30 | 44.4100751215 | 405 | 4.5e-112 |
| AEE88283.1 | CurG | BGC0000976 | Curacin\_biosynthetic\_gene\_cluster | 29 | 41.6703490941 | 405 | 4.5e-112 |
| AAT70102.1 | CurG | BGC0001165 | Curacin\_biosynthetic\_gene\_cluster | 29 | 41.6703490941 | 405 | 4.5e-112 |
| BAR73017.1 | putative\_PKS\_(KS-AT-KR-ACP-KS-AT-DH-KR-ACP) | BGC0001194 | Hitachimycin\_biosynthetic\_gene\_cluster | 31 | 44.0123729563 | 405 | 4.5e-112 |
| ACO94456.1 | polyketide\_synthase\_type\_I | BGC0000029 | BE-14106\_biosynthetic\_gene\_cluster | 30 | 41.7587273531 | 404 | 5.8e-112 |
| BAV32159.1 | polyketide\_synthase | BGC0001373 | Sordarin\_biosynthetic\_gene\_cluster | 30 | 46.3985859479 | 404 | 5.8e-112 |
| AEZ53946.1 | polyketide\_synthase | BGC0000144 | Salinomycin\_biosynthetic\_gene\_cluster | 32 | 40.8749447636 | 404 | 5.8e-112 |
| CAA16183.1 | polyketide\_synthase | BGC0001063 | Undecylprodigiosin\_biosynthetic\_gene\_cluster | 30 | 44.8519664163 | 404 | 7.6e-112 |
| BAR73007.1 | putative\_PKS\_(ACP-KS-AT-DH-KR-ACP-KS-AT-DH-ER-KR-ACP) | BGC0001194 | Hitachimycin\_biosynthetic\_gene\_cluster | 29 | 45.5148033584 | 404 | 9.9e-112 |
| BAH02270.1 | polyketide\_synthase | BGC0000126 | Pladienolide\_biosynthetic\_gene\_cluster | 31 | 40.8307556341 | 404 | 9.9e-112 |
| AAO62582.1 | polyketide\_synthase\_peptide\_sythetase\_fusion\_protein | BGC0001016 | Microcystin\_biosynthetic\_gene\_cluster | 30 | 41.3168360583 | 403 | 1.3e-111 |
| CAO85897.1 | modular\_polyketide\_synthase\_NorB | BGC0000110 | Neoaureothin\_biosynthetic\_gene\_cluster | 30 | 43.7914273089 | 403 | 1.3e-111 |
| AAF71767.1 | NysJ | BGC0000115 | Nystatin\_biosynthetic\_gene\_cluster | 32 | 39.8585947857 | 403 | 1.3e-111 |
| AFL48525.1 | laidlomycin\_polyketide\_synthase\_(loading\_module\_and\_module\_1) | BGC0000084 | Laidlomycin\_biosynthetic\_gene\_cluster | 32 | 40.4330534688 | 403 | 1.3e-111 |
| BAO66539.1 | type\_I\_polyketide\_synthase | BGC0000042 | Cremimycin\_biosynthetic\_gene\_cluster | 31 | 40.3888643394 | 403 | 1.7e-111 |
| ACY06289.1 | type\_I\_polyketide\_synthase | BGC0001042 | Sanglifehrin\_A\_biosynthetic\_gene\_cluster | 28 | 51.4361467079 | 403 | 2.2e-111 |
| AAP42859.1 | NanA5 | BGC0000105 | Nanchangmycin\_biosynthetic\_gene\_cluster | 31 | 44.5868316394 | 403 | 2.2e-111 |
| AEZ54379.1 | PieA6 | BGC0000124 | Piericidin\_A1\_biosynthetic\_gene\_cluster | 31 | 39.5050817499 | 403 | 2.2e-111 |
| ABC84459.1 | NigAIV | BGC0000114 | Nigericin\_biosynthetic\_gene\_cluster | 32 | 40.6098099867 | 403 | 2.2e-111 |
| AFL48520.1 | laidlomycin\_polyketide\_synthase\_(module\_9) | BGC0000084 | Laidlomycin\_biosynthetic\_gene\_cluster | 32 | 39.9911621741 | 403 | 2.2e-111 |
| AJO72736.1 | Type\_I\_modular\_polyketide\_synthase | BGC0001381 | Brasilinolide\_biosynthetic\_gene\_cluster | 30 | 43.3053468847 | 403 | 2.2e-111 |
| AAZ77673.1 | ChlB1 | BGC0000036 | Chlorothricin\_biosynthetic\_gene\_cluster | 31 | 40.2121078215 | 403 | 2.2e-111 |
| BAG85026.1 | putative\_polyketide\_synthase | BGC0000086 | Lasalocid\_biosynthetic\_gene\_cluster | 30 | 45.1171011931 | 402 | 2.9e-111 |
| CAD55506.1 | putative\_type\_I\_polyketide\_synthase | BGC0000038 | Coelimycin\_biosynthetic\_gene\_cluster | 29 | 46.3102076889 | 402 | 2.9e-111 |
| CAQ64686.1 | lasalocid\_modular\_polyketide\_synthase | BGC0000087 | Lasalocid\_biosynthetic\_gene\_cluster | 30 | 45.1171011931 | 402 | 2.9e-111 |
| ADC45534.1 | modular\_polyketide\_synthase | BGC0000093 | Meilingmycin\_biosynthetic\_gene\_cluster | 31 | 39.6818382678 | 402 | 3.8e-111 |
| ADB12490.1 | EpoC | BGC0000990 | Epothilone\_biosynthetic\_gene\_cluster | 29 | 45.9125055236 | 402 | 3.8e-111 |
| ABV83230.1 | CppC | BGC0000116 | Nystatin-like\_Pseudonocardia\_polyene\_biosynthetic\_gene\_cluster | 30 | 40.7865665046 | 401 | 6.4e-111 |
| AGI99496.1 | Type\_I\_polyketide\_synthase | BGC0001004 | Lobophorin\_biosynthetic\_gene\_cluster | 30 | 44.8519664163 | 401 | 6.4e-111 |
| AAF15892.2 | NosB | BGC0001028 | Nostopeptolide\_biosynthetic\_gene\_cluster | 30 | 40.3888643394 | 401 | 8.4e-111 |
| ALP32043.1 | CycC | BGC0001293 | Cyclizidine\_biosynthetic\_gene\_cluster | 30 | 39.5934600088 | 400 | 1.1e-110 |
| ctg1\_orf521 |  | BGC0001199 | Akaeolide\_biosynthetic\_gene\_cluster | 31 | 40.5656208573 | 400 | 1.4e-110 |
| AAP85335.1 | type\_I\_PKS | BGC0000233 | Hedamycin\_biosynthetic\_gene\_cluster | 29 | 44.9845338047 | 400 | 1.4e-110 |
| AAK73501.1 | AmphI | BGC0000015 | Amphotericin\_biosynthetic\_gene\_cluster | 30 | 39.7702165267 | 400 | 1.4e-110 |
| AAQ82566.1 | FscF | BGC0000061 | FR-008\_biosynthetic\_gene\_cluster | 32 | 40.1237295625 | 399 | 1.9e-110 |
| AAF62882.1 | EpoC | BGC0000991 | Epothilone\_biosynthetic\_gene\_cluster | 29 | 45.9125055236 | 399 | 1.9e-110 |
| CAJ88177.1 | putative\_type\_I\_polyketide\_synthase | BGC0000151 | Stambomycin\_biosynthetic\_gene\_cluster | 32 | 41.8029164825 | 399 | 1.9e-110 |
| CUW01176.1 | Type\_I\_polyketide\_synthase | BGC0001359\_c2 | PM100117\_/\_PM100118\_biosynthetic\_gene\_cluster | 30 | 40.9633230225 | 399 | 1.9e-110 |
| EFL02193.1 | amino\_acid\_adenylation\_domain-containing\_protein | BGC0000996 | Frontalamides\_biosynthetic\_gene\_cluster | 29 | 51.3919575784 | 399 | 3.2e-110 |
| CAI94713.1 | putative\_polyketide\_synthase | BGC0000141 | Rubradirin\_biosynthetic\_gene\_cluster | 30 | 39.9027839152 | 399 | 3.2e-110 |
| BAP34739.1 | type\_I\_polyketide\_synthase | BGC0000078 | Incednine\_biosynthetic\_gene\_cluster | 31 | 39.9027839152 | 398 | 4.2e-110 |
| ABP55210.1 | beta-ketoacyl\_synthase | BGC0000142 | Salinilactam\_biosynthetic\_gene\_cluster | 31 | 39.9911621741 | 398 | 5.4e-110 |
| BAF85843.1 | modular\_polyketide\_synthase | BGC0000109 | Nemadectin\_biosynthetic\_gene\_cluster | 30 | 46.0008837826 | 398 | 5.4e-110 |
| AAC01710.1 | RifA | BGC0000136 | Rifamycin\_biosynthetic\_gene\_cluster | 30 | 42.0680512594 | 398 | 5.4e-110 |
| CAC20931.1 | PimS1\_protein | BGC0000125 | Pimaricin\_biosynthetic\_gene\_cluster | 31 | 40.7423773752 | 398 | 7.1e-110 |
| AHH99925.1 | PKS\_I | BGC0000002 | Aculeximycin\_biosynthetic\_gene\_cluster | 32 | 39.4608926204 | 398 | 7.1e-110 |
| AAZ94386.1 | modular\_polyketide\_synthase | BGC0000040 | Concanamycin\_A\_biosynthetic\_gene\_cluster | 31 | 43.2611577552 | 398 | 7.1e-110 |
| BAE93729.1 | type\_I\_polyketide\_synthase | BGC0000164 | Tetronomycin\_biosynthetic\_gene\_cluster | 31 | 42.8192664605 | 398 | 7.1e-110 |
| ADA69241.1 | cis-AT\_polyketide\_synthase | BGC0001071 | Nosperin\_biosynthetic\_gene\_cluster | 29 | 40.3004860804 | 397 | 9.3e-110 |
| ACN69989.1 | polyketide\_synthase | BGC0000079 | Indanomycin\_biosynthetic\_gene\_cluster | 29 | 43.3053468847 | 397 | 9.3e-110 |
| CQR60496.1 | Polyketide\_synthase,\_type\_I,\_modules:\_4,\_5\_and\_6 | BGC0001287 | Chaxamycin\_A\_/\_chaxamycin\_B\_/\_chaxamycin\_C\_/\_chaxamycin\_D | 32 | 41.1400795404 | 397 | 1.2e-109 |
| AFV30250.1 | polyketide\_synthase | BGC0000075 | Hygrocin\_biosynthetic\_gene\_cluster | 31 | 40.2121078215 | 397 | 1.2e-109 |
| BAT51065.1 | type\_I\_polyketide\_synthase | BGC0001296 | Streptazone\_E\_biosynthetic\_gene\_cluster | 29 | 41.007512152 | 396 | 1.6e-109 |
| ADX66472.1 | ScnS1 | BGC0000108 | Natamycin\_biosynthetic\_gene\_cluster | 31 | 39.4167034909 | 396 | 1.6e-109 |
| ALP32042.1 | CycB | BGC0001293 | Cyclizidine\_biosynthetic\_gene\_cluster | 30 | 40.1237295625 | 396 | 1.6e-109 |
| ABB86410.1 | GelC | BGC0000067 | Geldanamycin\_biosynthetic\_gene\_cluster | 31 | 44.5426425099 | 396 | 2.1e-109 |
| ctg1\_orf7 |  | BGC0000053 | Elaiophylin\_biosynthetic\_gene\_cluster | 32 | 40.0353513036 | 396 | 2.7e-109 |
| CAD29795.1 | peptide\_synthetase | BGC0001015 | Microcystin\_biosynthetic\_gene\_cluster | 30 | 41.7145382236 | 396 | 2.7e-109 |
| ABI91466.1 | beta-ketoacyl\_synthase | BGC0001094 | Enacyloxin\_biosynthetic\_gene\_cluster | 32 | 39.9027839152 | 395 | 3.5e-109 |
| ABL86391.1 | hybrid\_polyketide\_synthase\_and\_nonribosomal\_peptide\_synthetase | BGC0000999 | Heat-stable\_antifungal\_factor\_biosynthetic\_gene\_cluster | 31 | 41.0517012815 | 395 | 3.5e-109 |
| AKD43522.1 | Type\_I\_polyketide\_synthase | BGC0001409 | Dutomycin\_biosynthetic\_gene\_cluster | 30 | 46.3985859479 | 395 | 4.6e-109 |
| ACO94470.1 | polyketide\_synthase\_type\_I | BGC0000029 | BE-14106\_biosynthetic\_gene\_cluster | 29 | 44.8961555457 | 395 | 4.6e-109 |
| BAH02268.1 | polyketide\_synthase | BGC0000126 | Pladienolide\_biosynthetic\_gene\_cluster | 31 | 40.7865665046 | 394 | 6e-109 |
| AAO06918.1 | GdmAIII | BGC0000066 | Geldanamycin\_biosynthetic\_gene\_cluster | 30 | 44.454264251 | 394 | 6e-109 |
| CAE02605.1 | polyketide\_synthase\_type\_I | BGC0000024 | Aureothin\_biosynthetic\_gene\_cluster | 31 | 39.9911621741 | 394 | 6e-109 |
| ABY83164.1 | Azi26 | BGC0000960 | Azinomycin\_B\_biosynthetic\_gene\_cluster | 29 | 44.0565620857 | 394 | 6e-109 |
| AHB82051.1 | polyketide\_synthase | BGC0001019 | Microsclerodermins\_biosynthetic\_gene\_cluster | 31 | 41.4052143173 | 394 | 6e-109 |
| ALA09371.1 | type\_I\_modular\_PKS | BGC0001303 | Lobosamide\_biosynthetic\_gene\_cluster | 28 | 45.0287229342 | 394 | 7.9e-109 |
| ctg1\_orf522 |  | BGC0001199 | Akaeolide\_biosynthetic\_gene\_cluster | 29 | 44.5426425099 | 394 | 7.9e-109 |
| CAE02606.1 | polyketide\_synthase\_type\_I | BGC0000024 | Aureothin\_biosynthetic\_gene\_cluster | 31 | 40.1237295625 | 394 | 1e-108 |
| ACB37743.1 | putative\_type\_I\_polyketide\_synthase | BGC0000162 | Tetrocarcin\_A\_biosynthetic\_gene\_cluster | 31 | 40.5214317278 | 393 | 1.3e-108 |
| CAQ64692.1 | lasalocid\_modular\_polyketide\_synthase | BGC0000087 | Lasalocid\_biosynthetic\_gene\_cluster | 32 | 39.7260273973 | 393 | 1.8e-108 |
| AAQ84145.1 | Plm5 | BGC0000123 | Phoslactomycin\_B\_biosynthetic\_gene\_cluster | 30 | 45.1612903226 | 393 | 1.8e-108 |
| AAQ90174.1 | polyketide\_synthase\_type\_I | BGC0000128 | Pyoluteorin\_biosynthetic\_gene\_cluster | 30 | 41.4052143173 | 393 | 1.8e-108 |
| AGI99497.1 | type\_I\_polyketide\_synthase | BGC0001004 | Lobophorin\_biosynthetic\_gene\_cluster | 30 | 40.2121078215 | 393 | 2.3e-108 |
| ABV97152.1 | Beta-ketoacyl\_synthase | BGC0000137 | Rifamycin\_biosynthetic\_gene\_cluster | 32 | 40.6098099867 | 393 | 2.3e-108 |
| CUW01171.1 | Type\_I\_polyketide\_synthase | BGC0001359 | PM100117\_/\_PM100118\_biosynthetic\_gene\_cluster | 30 | 42.0680512594 | 393 | 2.3e-108 |
| AHB82059.1 | non\_ribosomal\_peptide\_synthetase/polyketide\_synthase | BGC0001019 | Microsclerodermins\_biosynthetic\_gene\_cluster | 31 | 40.7865665046 | 392 | 3.9e-108 |
| BAG85030.1 | putative\_polyketide\_synthase | BGC0000086 | Lasalocid\_biosynthetic\_gene\_cluster | 32 | 39.5934600088 | 392 | 3.9e-108 |
| CAQ64690.1 | lasalocid\_modular\_polyketide\_synthase | BGC0000087 | Lasalocid\_biosynthetic\_gene\_cluster | 32 | 39.5934600088 | 392 | 3.9e-108 |
| BAG17643.1 | putative\_NRPS-type-I\_PKS\_fusion\_protein | BGC0001043 | SGR\_PTMs\_biosynthetic\_gene\_cluster | 31 | 45.1171011931 | 392 | 3.9e-108 |
| ACZ65476.1 | type\_I\_modular\_polyketide\_synthase | BGC0000140 | RK-682\_biosynthetic\_gene\_cluster | 31 | 38.9306230667 | 392 | 3.9e-108 |
| ABK32255.1 | AmbA | BGC0000014 | Ambruticin\_biosynthetic\_gene\_cluster | 30 | 44.4100751215 | 391 | 5.1e-108 |
| AIT55264.1 | GulA | BGC0000072 | Gulmirecin\_biosynthetic\_gene\_cluster | 32 | 39.6376491383 | 391 | 5.1e-108 |
| AHH99926.1 | PKS\_I | BGC0000002 | Aculeximycin\_biosynthetic\_gene\_cluster | 29 | 44.6310207689 | 391 | 5.1e-108 |
| AGC09484.1 | LobS1 | BGC0001183 | Lobophorin\_biosynthetic\_gene\_cluster | 30 | 40.1237295625 | 391 | 8.7e-108 |
| CBD77746.1 | non-ribosomal\_peptide\_synthetase/polyketide\_synthase | BGC0000974 | Crocacin\_biosynthetic\_gene\_cluster | 31 | 39.9027839152 | 391 | 8.7e-108 |
| CQR60497.1 | Polyketide\_synthase,\_type\_I,\_modules:\_loading,\_1,\_2\_and\_3 | BGC0001287 | Chaxamycin\_A\_/\_chaxamycin\_B\_/\_chaxamycin\_C\_/\_chaxamycin\_D | 29 | 43.0844012373 | 391 | 8.7e-108 |
| ABV83229.1 | CppB | BGC0000116 | Nystatin-like\_Pseudonocardia\_polyene\_biosynthetic\_gene\_cluster | 31 | 39.9469730446 | 391 | 8.7e-108 |
| ACO94498.1 | polyketide\_synthase\_type\_I | BGC0000097 | ML-449\_biosynthetic\_gene\_cluster | 30 | 43.1285903668 | 391 | 8.7e-108 |
| BAC20566.1 | polyketide\_synthase | BGC0000039 | Compactin\_biosynthetic\_gene\_cluster | 30 | 43.9681838268 | 391 | 8.7e-108 |
| AAC01713.1 | RifD | BGC0000136 | Rifamycin\_biosynthetic\_gene\_cluster | 31 | 39.8585947857 | 390 | 1.1e-107 |
| AEU17899.1 | putative\_type\_I\_PKS | BGC0001072 | Simocyclinone\_biosynthetic\_gene\_cluster | 30 | 41.4052143173 | 390 | 1.5e-107 |
| ABK32287.1 | JerA | BGC0000080 | Jerangolid\_biosynthetic\_gene\_cluster | 31 | 40.5656208573 | 389 | 1.9e-107 |
| ABV97153.1 | Beta-ketoacyl\_synthase | BGC0000137 | Rifamycin\_biosynthetic\_gene\_cluster | 31 | 39.8585947857 | 389 | 1.9e-107 |
| ACB46194.1 | polyketide\_synthase | BGC0000989 | Epothilone\_biosynthetic\_gene\_cluster | 29 | 45.9566946531 | 389 | 1.9e-107 |
| BAO66541.1 | type\_I\_polyketide\_synthase | BGC0000042 | Cremimycin\_biosynthetic\_gene\_cluster | 30 | 39.5934600088 | 389 | 1.9e-107 |
| EAU29529.1 | hypothetical\_protein | BGC0000682 | Terretonin\_biosynthetic\_gene\_cluster | 26 | 67.5209898365 | 389 | 2.5e-107 |
| BAC57028.1 | protomycinolide\_IV\_synthase\_1 | BGC0000102 | Mycinamicin\_biosynthetic\_gene\_cluster | 31 | 41.3610251878 | 389 | 3.3e-107 |
| ABJ97439.1 | MerC | BGC0001012 | Meridamycin\_biosynthetic\_gene\_cluster | 29 | 45.6915598763 | 388 | 4.3e-107 |
| ACR50773.1 | polyketide\_synthase | BGC0000163 | Tetronasin\_biosynthetic\_gene\_cluster | 30 | 45.6031816173 | 388 | 4.3e-107 |
| BAC68125.1 | modular\_polyketide\_synthase | BGC0000059 | Filipin\_biosynthetic\_gene\_cluster | 30 | 41.9796730004 | 388 | 5.6e-107 |
| AAQ82568.1 | FscD | BGC0000034 | Candicidin\_biosynthetic\_gene\_cluster | 30 | 40.7865665046 | 388 | 5.6e-107 |
| CAQ34929.1 | putative\_polyketide\_synthase | BGC0000986 | Dkxanthene\_biosynthetic\_gene\_cluster | 32 | 40.1237295625 | 388 | 5.6e-107 |
| AEZ54378.1 | PieA5 | BGC0000124 | Piericidin\_A1\_biosynthetic\_gene\_cluster | 31 | 40.5214317278 | 388 | 7.4e-107 |
| WP\_043476516.1 | beta-ketoacyl\_synthase | BGC0001232 | Leucanicidin\_biosynthetic\_gene\_cluster | 31 | 40.3446752099 | 387 | 9.6e-107 |
| ABC84469.1 | NigAIX | BGC0000114 | Nigericin\_biosynthetic\_gene\_cluster | 30 | 40.3888643394 | 387 | 9.6e-107 |
| ADH04657.1 | TugA | BGC0001342 | Thuggacin\_biosynthetic\_gene\_cluster | 32 | 38.7096774194 | 387 | 9.6e-107 |
| CAE45669.1 | borrelidin\_polyketide\_synthase,\_type\_I | BGC0000031 | Borrelidin\_biosynthetic\_gene\_cluster | 29 | 47.3707467963 | 387 | 1.3e-106 |
| CAE45671.1 | borrelidin\_polyketide\_synthase,\_type\_I | BGC0000031 | Borrelidin\_biosynthetic\_gene\_cluster | 30 | 39.4167034909 | 387 | 1.3e-106 |
| ABW96542.1 | type\_I\_modular\_polyketide\_synthase | BGC0000159 | Tautomycin\_biosynthetic\_gene\_cluster | 29 | 45.4264250994 | 387 | 1.3e-106 |
| AEK75502.1 | type\_1\_polyketide\_synthase | BGC0000001 | Abyssomicin\_biosynthetic\_gene\_cluster | 31 | 42.6425099426 | 387 | 1.3e-106 |
| BAO66519.1 | type\_I\_polyketide\_synthase | BGC0000042 | Cremimycin\_biosynthetic\_gene\_cluster | 30 | 40.256296951 | 386 | 1.6e-106 |
| AKL78824.1 | GLPKS3 | BGC0001187 | Xenolozoyenone\_biosynthetic\_gene\_cluster | 30 | 41.1400795404 | 386 | 1.6e-106 |
| AAC01711.1 | RifB | BGC0000136 | Rifamycin\_biosynthetic\_gene\_cluster | 31 | 39.8144056562 | 386 | 1.6e-106 |
| AEZ53945.1 | polyketide\_synthase | BGC0000144 | Salinomycin\_biosynthetic\_gene\_cluster | 31 | 40.256296951 | 386 | 1.6e-106 |
| CAJ88186.1 | putative\_modular\_polyketide\_synthase | BGC0000151 | Stambomycin\_biosynthetic\_gene\_cluster | 32 | 39.6818382678 | 386 | 2.1e-106 |
| AEZ53952.1 | polyketide\_synthase | BGC0000144 | Salinomycin\_biosynthetic\_gene\_cluster | 31 | 42.0680512594 | 386 | 2.1e-106 |
| AGY30677.1 | Ann5 | BGC0001298 | 4-Z-annimycin\_biosynthetic\_gene\_cluster | 27 | 52.187361909 | 386 | 2.1e-106 |
| ctg1\_orf11 |  | BGC0000053 | Elaiophylin\_biosynthetic\_gene\_cluster | 29 | 46.3985859479 | 386 | 2.8e-106 |
| ctg1\_orf0002 |  | BGC0001068 | Pyripyropene\_A\_biosynthetic\_gene\_cluster | 31 | 46.3985859479 | 386 | 2.8e-106 |
| AFU82614.1 | mixed\_NRPS\_PKS | BGC0000998 | Guadinomine\_biosynthetic\_gene\_cluster | 30 | 40.3004860804 | 386 | 2.8e-106 |
| AAP85336.1 | type\_I\_PKS | BGC0000233 | Hedamycin\_biosynthetic\_gene\_cluster | 30 | 40.3888643394 | 385 | 4.8e-106 |
| CAJ88184.1 | putative\_modular\_polyketide\_synthase | BGC0000151 | Stambomycin\_biosynthetic\_gene\_cluster | 31 | 39.328325232 | 385 | 4.8e-106 |
| ADM46356.1 | polyketide\_synthase | BGC0000106 | Naphthomycin\_biosynthetic\_gene\_cluster | 31 | 42.2448077773 | 385 | 4.8e-106 |
| ADZ24995.1 | non-ribosomal\_peptide\_synthase/polyketide\_synthase | BGC0000380 | Leupyrrin\_biosynthetic\_gene\_cluster | 30 | 44.1007512152 | 384 | 6.2e-106 |
| CAJ88175.1 | putative\_polyketide\_synthase\_B | BGC0000151 | Stambomycin\_biosynthetic\_gene\_cluster | 31 | 39.8144056562 | 384 | 6.2e-106 |
| AMK48247.1 | 6-methylsalicylic\_acid\_synthase | BGC0001351\_c2 | Cahuitamycins\_biosynthetic\_gene\_cluster | 31 | 41.4935925762 | 384 | 8.1e-106 |
| CAO98852.1 | polyketide\_synthase\_AufI | BGC0000023 | Aurafuron\_biosynthetic\_gene\_cluster | 32 | 39.7260273973 | 384 | 8.1e-106 |
| CAQ52624.1 | type\_I\_polyketide\_synthase,\_modules\_7-8 | BGC0001066 | Kendomycin\_biosynthetic\_gene\_cluster | 27 | 51.0384445426 | 384 | 8.1e-106 |
| ACN69992.1 | polyketide\_synthase | BGC0000079 | Indanomycin\_biosynthetic\_gene\_cluster | 30 | 40.4330534688 | 384 | 8.1e-106 |
| CAD15508.1 | probable\_polyketide\_synthase\_protein | BGC0001014 | Micacocidin\_biosynthetic\_gene\_cluster | 31 | 40.7423773752 | 384 | 8.1e-106 |
| CAI94682.1 | putative\_polyketide\_synthase | BGC0000141 | Rubradirin\_biosynthetic\_gene\_cluster | 30 | 40.7423773752 | 384 | 1.1e-105 |
| ACF35445.1 | MbcAI | BGC0000090 | Macbecin\_biosynthetic\_gene\_cluster | 30 | 40.167918692 | 384 | 1.1e-105 |
| AHB82072.1 | non\_ribosomal\_peptide\_synthetase/polyketide\_synthase | BGC0001231 | Microsclerodermins\_biosynthetic\_gene\_cluster | 31 | 42.3773751657 | 384 | 1.1e-105 |
| AGY62755.1 | EbeC | BGC0000051 | Ebelactone\_biosynthetic\_gene\_cluster | 29 | 39.7702165267 | 383 | 1.4e-105 |
| BAG85032.1 | putative\_polyketide\_synthase | BGC0000086 | Lasalocid\_biosynthetic\_gene\_cluster | 31 | 39.3725143615 | 383 | 1.4e-105 |
| BAO66543.1 | type\_I\_polyketide\_synthase | BGC0000042 | Cremimycin\_biosynthetic\_gene\_cluster | 29 | 39.6376491383 | 383 | 1.8e-105 |
| CBZ41585.1 | putative\_modular\_polyketide\_synthase | BGC0000151 | Stambomycin\_biosynthetic\_gene\_cluster | 30 | 43.3937251436 | 383 | 2.4e-105 |
| ABB88520.1 | polyketide\_synthase\_type\_I | BGC0000050 | E-837\_biosynthetic\_gene\_cluster | 30 | 45.6915598763 | 382 | 3.1e-105 |
| AAF71774.1 | NysA | BGC0000115 | Nystatin\_biosynthetic\_gene\_cluster | 31 | 39.7260273973 | 382 | 3.1e-105 |
| CAQ34918.1 | nonribosomal\_peptide\_synthetase/\_polyketide\_synthase | BGC0000986 | Dkxanthene\_biosynthetic\_gene\_cluster | 28 | 44.8961555457 | 382 | 4e-105 |
| AJW65409.1 | type\_I\_modular\_polyketide\_synthase | BGC0001195 | Nocardiopsin\_biosynthetic\_gene\_cluster | 31 | 40.0795404331 | 381 | 5.3e-105 |
| AAD03048.1 | type\_I\_polyketide\_synthase | BGC0000041 | Coronafacic\_acid\_biosynthetic\_gene\_cluster | 28 | 45.4706142289 | 381 | 5.3e-105 |
| AEU17898.1 | putative\_type\_I\_PKS | BGC0001072 | Simocyclinone\_biosynthetic\_gene\_cluster | 30 | 40.5656208573 | 381 | 6.9e-105 |
| CAP95404.1 |  | BGC0001404 | Sorbicillin\_biosynthetic\_gene\_cluster | 28 | 52.4083075563 | 381 | 6.9e-105 |
| CQR60495.1 | Polyketide\_synthase,\_type\_I,\_module\_7 | BGC0001287 | Chaxamycin\_A\_/\_chaxamycin\_B\_/\_chaxamycin\_C\_/\_chaxamycin\_D | 30 | 39.6376491383 | 381 | 6.9e-105 |
| AHB82062.1 | polyketide\_synthase | BGC0001231 | Microsclerodermins\_biosynthetic\_gene\_cluster | 30 | 40.3888643394 | 381 | 9e-105 |
| ADC79618.1 | BafAIII | BGC0000028 | Bafilomycin\_biosynthetic\_gene\_cluster | 28 | 47.5033141847 | 381 | 9e-105 |
| ctg1\_orf9 |  | BGC0000053 | Elaiophylin\_biosynthetic\_gene\_cluster | 30 | 39.7260273973 | 381 | 9e-105 |
| AAQ84157.1 | Plm2-3 | BGC0000123 | Phoslactomycin\_B\_biosynthetic\_gene\_cluster | 30 | 40.3446752099 | 381 | 9e-105 |
| ACF35446.1 | MbcAII | BGC0000090 | Macbecin\_biosynthetic\_gene\_cluster | 32 | 39.7702165267 | 380 | 1.2e-104 |
| AIA58899.1 | HRPKS | BGC0001141 | Brefeldin\_biosynthetic\_gene\_cluster | 32 | 42.4657534247 | 380 | 1.2e-104 |
| EHA28244.1 | hypothetical\_protein | BGC0001143 | Azanigerone\_biosynthetic\_gene\_cluster | 30 | 43.5704816615 | 380 | 1.5e-104 |
| ABB05102.1 | LipPks1 | BGC0001003 | Lipomycin\_biosynthetic\_gene\_cluster | 30 | 40.6098099867 | 380 | 1.5e-104 |
| AEZ54375.1 | PieA2 | BGC0000124 | Piericidin\_A1\_biosynthetic\_gene\_cluster | 31 | 40.6098099867 | 380 | 1.5e-104 |
| AAQ84156.1 | Plm1 | BGC0000123 | Phoslactomycin\_B\_biosynthetic\_gene\_cluster | 29 | 39.9469730446 | 379 | 2e-104 |
| AGC95324.1 | CurS1 | BGC0000045 | Dehydrocurvularin\_biosynthetic\_gene\_cluster | 28 | 54.6619531595 | 379 | 3.4e-104 |
| AHH99922.1 | PKS\_I | BGC0000002 | Aculeximycin\_biosynthetic\_gene\_cluster | 30 | 40.6539991162 | 379 | 3.4e-104 |
| ALA09355.1 | type\_I\_modular\_PKS | BGC0001303 | Lobosamide\_biosynthetic\_gene\_cluster | 30 | 39.4608926204 | 378 | 4.5e-104 |
| ABV97151.1 | AMP-dependent\_synthetase\_and\_ligase | BGC0000137 | Rifamycin\_biosynthetic\_gene\_cluster | 31 | 42.9076447194 | 378 | 4.5e-104 |
| BAE93730.1 | type\_I\_polyketide\_synthase | BGC0000164 | Tetronomycin\_biosynthetic\_gene\_cluster | 30 | 44.4100751215 | 378 | 4.5e-104 |
| CAE45670.1 | borrelidin\_polyketide\_synthase,\_type\_I | BGC0000031 | Borrelidin\_biosynthetic\_gene\_cluster | 30 | 39.328325232 | 378 | 4.5e-104 |
| AAC69331.1 | type\_I\_polyketide\_synthase\_PikAIII | BGC0000094 | Methymycin\_/\_pikromycin\_biosynthetic\_gene\_cluster | 30 | 40.5656208573 | 378 | 5.8e-104 |
| AAK57190.1 | MxaF | BGC0001022 | Myxalamid\_biosynthetic\_gene\_cluster | 30 | 39.0190013257 | 378 | 7.6e-104 |
| AKD43761.1 | HerD | BGC0001349 | Heronamide\_biosynthetic\_gene\_cluster | 30 | 40.1237295625 | 378 | 7.6e-104 |
| ABI93779.1 | GdmPKS | BGC0000068 | Geldanamycin\_biosynthetic\_gene\_cluster | 30 | 41.095890411 | 378 | 7.6e-104 |
| AKD43764.1 | HerF | BGC0001349 | Heronamide\_biosynthetic\_gene\_cluster | 31 | 39.1515687141 | 377 | 1e-103 |
| CAQ18839.1 | hybrid\_polyketide\_synthase/nonribosomal\_polypetide\_synthetase | BGC0000954 | Ajudazol\_biosynthetic\_gene\_cluster | 30 | 44.4100751215 | 377 | 1e-103 |
| AAY28227.1 | HbmAIII | BGC0000074 | Herbimycin\_biosynthetic\_gene\_cluster | 29 | 40.6098099867 | 377 | 1e-103 |
| AAM70355.1 | CalO5 | BGC0000033 | Calicheamicin\_biosynthetic\_gene\_cluster | 29 | 44.1891294741 | 377 | 1e-103 |
| AGZ15474.1 | putative\_type\_I\_polyketide\_synthase | BGC0001036 | Polyoxypeptin\_biosynthetic\_gene\_cluster | 28 | 44.0565620857 | 377 | 1.3e-103 |
| AHH99923.1 | PKS\_I | BGC0000002 | Aculeximycin\_biosynthetic\_gene\_cluster | 31 | 39.6376491383 | 377 | 1.3e-103 |
| ctg1\_orf523 |  | BGC0001199 | Akaeolide\_biosynthetic\_gene\_cluster | 30 | 40.167918692 | 376 | 1.7e-103 |
| ABC87512.1 | polyketide\_synthase | BGC0001011 | Meridamycin\_biosynthetic\_gene\_cluster | 28 | 45.7357490057 | 376 | 2.2e-103 |
| ctg1\_orf23 |  | BGC0001013 | Meridamycin\_biosynthetic\_gene\_cluster | 28 | 45.7357490057 | 376 | 2.2e-103 |
| AAF86396.1 | FkbA | BGC0000994 | FK520\_biosynthetic\_gene\_cluster | 32 | 39.8144056562 | 376 | 2.2e-103 |
| AAC01712.2 | RifC | BGC0000136 | Rifamycin\_biosynthetic\_gene\_cluster | 30 | 39.4167034909 | 376 | 2.9e-103 |
| ADC79638.1 | TamAII | BGC0001052 | Tirandamycin\_biosynthetic\_gene\_cluster | 29 | 41.007512152 | 376 | 2.9e-103 |
| ABB88519.1 | polyketide\_synthase\_type\_I | BGC0000050 | E-837\_biosynthetic\_gene\_cluster | 31 | 41.095890411 | 375 | 3.8e-103 |
| ACY06287.1 | type\_I\_polyketide\_synthase | BGC0001042 | Sanglifehrin\_A\_biosynthetic\_gene\_cluster | 31 | 39.5492708794 | 375 | 3.8e-103 |
| ACO94471.1 | polyketide\_synthase\_type\_I | BGC0000029 | BE-14106\_biosynthetic\_gene\_cluster | 30 | 40.256296951 | 375 | 4.9e-103 |
| ABV83228.1 | CppA | BGC0000116 | Nystatin-like\_Pseudonocardia\_polyene\_biosynthetic\_gene\_cluster | 31 | 39.7260273973 | 375 | 4.9e-103 |
| AHB82052.1 | polyketide\_synthase | BGC0001019 | Microsclerodermins\_biosynthetic\_gene\_cluster | 36 | 29.2532037119 | 375 | 4.9e-103 |
| AHB82063.1 | polyketide\_synthase | BGC0001231 | Microsclerodermins\_biosynthetic\_gene\_cluster | 36 | 29.4741493593 | 375 | 4.9e-103 |
| AAC69330.1 | type\_I\_polyketide\_synthase\_PikAII | BGC0000094 | Methymycin\_/\_pikromycin\_biosynthetic\_gene\_cluster | 31 | 40.0795404331 | 374 | 6.5e-103 |
| ABC84470.1 | NIGAVIII | BGC0000114 | Nigericin\_biosynthetic\_gene\_cluster | 29 | 44.0123729563 | 374 | 6.5e-103 |
| ACB46486.1 | polyketide\_synthase | BGC0000082 | Kijanimicin\_biosynthetic\_gene\_cluster | 29 | 45.38223597 | 374 | 6.5e-103 |
| ABC84460.1 | NigAV | BGC0000114 | Nigericin\_biosynthetic\_gene\_cluster | 31 | 41.4052143173 | 374 | 6.5e-103 |
| ADH04639.1 | TgaA | BGC0001051 | Thuggacin\_biosynthetic\_gene\_cluster | 32 | 39.3725143615 | 374 | 6.5e-103 |
| BAE93731.1 | type\_I\_polyketide\_synthase | BGC0000164 | Tetronomycin\_biosynthetic\_gene\_cluster | 29 | 46.1776403005 | 374 | 6.5e-103 |
| AAD03047.1 | type\_I\_polyketide\_synthase | BGC0000041 | Coronafacic\_acid\_biosynthetic\_gene\_cluster | 28 | 47.8568272205 | 374 | 8.4e-103 |
| CBZ41586.1 | putative\_modular\_polyketide\_synthase | BGC0000151 | Stambomycin\_biosynthetic\_gene\_cluster | 31 | 40.167918692 | 374 | 1.1e-102 |
| BAQ25507.1 | type\_I\_polyketide\_synthase | BGC0001288 | Maklamicin\_biosynthetic\_gene\_cluster | 31 | 39.9911621741 | 374 | 1.1e-102 |
| ctg1\_orf29 |  | BGC0000096 | Midecamycin\_biosynthetic\_gene\_cluster | 29 | 45.2054794521 | 374 | 1.1e-102 |
| AAM77986.1 | iterative\_type\_I\_polyketide\_synthase | BGC0000112 | Neocarzinostatin\_biosynthetic\_gene\_cluster | 29 | 43.7472381794 | 374 | 1.1e-102 |
| CAM00064.1 | EryAII\_Erythromycin\_polyketide\_synthase\_modules\_3\_and\_4 | BGC0000055 | Erythromycin\_biosynthetic\_gene\_cluster | 28 | 48.6964206805 | 373 | 1.4e-102 |
| CQR60494.1 | Polyketide\_synthase,\_type\_I,\_module\_8 | BGC0001287 | Chaxamycin\_A\_/\_chaxamycin\_B\_/\_chaxamycin\_C\_/\_chaxamycin\_D | 30 | 41.5377817057 | 373 | 1.4e-102 |
| ADM46357.1 | polyketide\_synthase | BGC0000106 | Naphthomycin\_biosynthetic\_gene\_cluster | 29 | 44.454264251 | 373 | 1.4e-102 |
| AFD30954.1 | CrmA | BGC0000966 | Caerulomycin\_A\_biosynthetic\_gene\_cluster | 31 | 41.0517012815 | 373 | 2.5e-102 |
| ABY66019.1 | 6-methylsalicylic\_acid\_synthase | BGC0001008 | Maduropeptin\_biosynthetic\_gene\_cluster | 33 | 40.3446752099 | 373 | 2.5e-102 |
| AGC24271.1 | PrlQ | BGC0001038 | Pyralomicin\_biosynthetic\_gene\_cluster | 32 | 40.256296951 | 372 | 3.2e-102 |
| CAL58681.1 | polyketide\_synthase | BGC0000149 | Spirangien\_biosynthetic\_gene\_cluster | 30 | 41.847105612 | 372 | 4.2e-102 |
| CAQ52623.1 | type\_I\_polyketide\_synthase,\_module\_6 | BGC0001066 | Kendomycin\_biosynthetic\_gene\_cluster | 28 | 45.38223597 | 371 | 5.5e-102 |
| CBA11583.1 | polyketide\_synthase\_type\_I | BGC0001046 | Streptolydigin\_biosynthetic\_gene\_cluster | 29 | 39.9469730446 | 371 | 5.5e-102 |
| ABB86408.1 | GelA | BGC0000067 | Geldanamycin\_biosynthetic\_gene\_cluster | 27 | 52.2757401679 | 371 | 9.3e-102 |
| AAO06916.1 | GdmAI | BGC0000066 | Geldanamycin\_biosynthetic\_gene\_cluster | 30 | 43.9681838268 | 371 | 9.3e-102 |
| AEC13070.1 | fosD | BGC0000060 | Fostriecin\_biosynthetic\_gene\_cluster | 31 | 41.3168360583 | 371 | 9.3e-102 |
| ABB88534.1 | polyketide\_synthase\_type\_I | BGC0000049 | E-492\_/\_E-975\_biosynthetic\_gene\_cluster | 30 | 44.8077772868 | 371 | 9.3e-102 |
| ADU85988.1 | putative\_iterative\_type\_I\_polyketide\_synthase | BGC0000165 | Tiacumicin\_B\_biosynthetic\_gene\_cluster | 30 | 39.8585947857 | 369 | 2.1e-101 |
| KGO40478.1 | Acyl\_transferase/acyl\_hydrolase/lysophospholipase | BGC0001205 | Communesin\_biosynthetic\_gene\_cluster | 30 | 42.3331860362 | 369 | 2.7e-101 |
| ACB37740.1 | putative\_type\_I\_polyketide\_synthase | BGC0000162 | Tetrocarcin\_A\_biosynthetic\_gene\_cluster | 28 | 49.0499337163 | 369 | 2.7e-101 |
| ABV91286.1 | type\_I\_modular\_polyketide\_synthase | BGC0000158 | Tautomycetin\_biosynthetic\_gene\_cluster | 30 | 39.9027839152 | 369 | 3.5e-101 |
| ADC45586.1 | modular\_polyketide\_synthase | BGC0000093 | Meilingmycin\_biosynthetic\_gene\_cluster | 28 | 44.9845338047 | 368 | 4.6e-101 |
| CAA60462.1 | polyketide\_synthase | BGC0001040 | Rapamycin\_biosynthetic\_gene\_cluster | 30 | 41.6703490941 | 368 | 6e-101 |
| AHB82070.1 | polyketide\_synthase | BGC0001231 | Microsclerodermins\_biosynthetic\_gene\_cluster | 29 | 41.8029164825 | 368 | 7.9e-101 |
| BAG85029.1 | putative\_polyketide\_synthase | BGC0000086 | Lasalocid\_biosynthetic\_gene\_cluster | 30 | 40.1237295625 | 368 | 7.9e-101 |
| ABI94379.1 | tautomycetin\_biosynthetic\_PKS | BGC0000157 | Tautomycetin\_biosynthetic\_gene\_cluster | 30 | 39.9027839152 | 368 | 7.9e-101 |
| AJW65407.1 | type\_I\_modular\_polyketide\_synthase | BGC0001195 | Nocardiopsin\_biosynthetic\_gene\_cluster | 30 | 40.8749447636 | 367 | 1e-100 |
| AAP42874.1 | NanA8 | BGC0000105 | Nanchangmycin\_biosynthetic\_gene\_cluster | 29 | 40.167918692 | 367 | 1.3e-100 |
| AEK75504.1 | type\_1\_polyketide\_synthase | BGC0000001 | Abyssomicin\_biosynthetic\_gene\_cluster | 30 | 39.9027839152 | 367 | 1.3e-100 |
| ACR50785.1 | polyketide\_synthase | BGC0000163 | Tetronasin\_biosynthetic\_gene\_cluster | 30 | 41.1400795404 | 367 | 1.3e-100 |
| CAQ64689.1 | lasalocid\_modular\_polyketide\_synthase | BGC0000087 | Lasalocid\_biosynthetic\_gene\_cluster | 30 | 40.1237295625 | 367 | 1.3e-100 |
| AAC46026.1 | polyketide\_synthase\_modules\_4\_and\_5 | BGC0000113 | Niddamycin\_biosynthetic\_gene\_cluster | 28 | 52.9827662395 | 366 | 1.8e-100 |
| ADC79617.1 | BafAII | BGC0000028 | Bafilomycin\_biosynthetic\_gene\_cluster | 31 | 40.8307556341 | 366 | 3e-100 |
| EHA19289.1 | hypothetical\_protein | BGC0001124 | Pyranonigrin\_E\_biosynthetic\_gene\_cluster | 27 | 50.0662836942 | 366 | 3e-100 |
| AAS98200.1 | MSAS-type\_polyketide\_synthase | BGC0001273 | Asperlactone\_biosynthetic\_gene\_cluster | 29 | 41.3168360583 | 366 | 3e-100 |
| AAY28225.1 | HbmAI | BGC0000074 | Herbimycin\_biosynthetic\_gene\_cluster | 30 | 44.0123729563 | 365 | 3.9e-100 |
| ACB46471.1 | polyketide\_synthase | BGC0000082 | Kijanimicin\_biosynthetic\_gene\_cluster | 28 | 46.4427750773 | 365 | 5.1e-100 |
| AEP40940.1 | polyketide\_synthase\_type\_I | BGC0000021 | Apoptolidin\_biosynthetic\_gene\_cluster | 30 | 40.167918692 | 364 | 6.7e-100 |
| CAQ43079.1 | polyketide\_synthase | BGC0000970 | Chondrochloren\_biosynthetic\_gene\_cluster | 30 | 41.9796730004 | 364 | 6.7e-100 |
| ACY06288.1 | type\_I\_polyketide\_synthase | BGC0001042 | Sanglifehrin\_A\_biosynthetic\_gene\_cluster | 31 | 39.8585947857 | 364 | 6.7e-100 |
| ADC45516.1 | modular\_polyketide\_synthase | BGC0000093 | Meilingmycin\_biosynthetic\_gene\_cluster | 30 | 39.4167034909 | 363 | 1.5e-99 |
| EAL89230.2 | LovB-like\_polyketide\_synthase,\_putative | BGC0000129 | Pyripyropene\_biosynthetic\_gene\_cluster | 30 | 41.0517012815 | 363 | 1.9e-99 |
| WP\_043476519.1 | polyketide\_synthase | BGC0001232 | Leucanicidin\_biosynthetic\_gene\_cluster | 31 | 40.6098099867 | 362 | 3.3e-99 |
| CAF05651.1 | TubF\_protein | BGC0001053 | Tubulysin\_biosynthetic\_gene\_cluster | 32 | 39.9911621741 | 362 | 4.3e-99 |
| ACO94499.1 | polyketide\_synthase\_type\_I | BGC0000097 | ML-449\_biosynthetic\_gene\_cluster | 30 | 40.3446752099 | 361 | 5.7e-99 |
| EAQ86392.1 | hypothetical\_protein | BGC0001405 | Chaetoviridin\_/\_chaetomugilin\_biosynthetic\_gene\_cluster | 32 | 36.5002209456 | 361 | 7.4e-99 |
| AHV78252.1 | ResS1 | BGC0001246 | Trans-resorcylide\_biosynthetic\_gene\_cluster | 31 | 41.1400795404 | 360 | 1.6e-98 |
| EYT83439.1 | beta-ketoacyl\_synthase | BGC0001213 | Nataxazole\_biosynthetic\_gene\_cluster | 31 | 38.6654882899 | 359 | 3.7e-98 |
| AAU93807.2 | polyketide\_synthase\_modules\_1\_and\_2 | BGC0000054 | Erythromycin\_biosynthetic\_gene\_cluster | 29 | 39.7702165267 | 359 | 3.7e-98 |
| ACD39758.1 | reducing\_polyketide\_synthase | BGC0000076 | Hypothemycin\_biosynthetic\_gene\_cluster | 31 | 41.095890411 | 358 | 6.2e-98 |
| AAY28226.1 | HbmAII | BGC0000074 | Herbimycin\_biosynthetic\_gene\_cluster | 29 | 40.3888643394 | 358 | 6.2e-98 |
| ACD39767.1 | reducing\_polyketide\_synthase | BGC0000077 | Hypothemycin\_biosynthetic\_gene\_cluster | 31 | 41.095890411 | 358 | 6.2e-98 |
| ABB88523.1 | polyketide\_synthase\_type\_I | BGC0000050 | E-837\_biosynthetic\_gene\_cluster | 31 | 40.9191338931 | 358 | 8.2e-98 |
| AAX98190.1 | polyketide\_synthase\_type\_I | BGC0000052 | ECO-02301\_biosynthetic\_gene\_cluster | 28 | 42.0238621299 | 358 | 8.2e-98 |
| BAJ16471.1 | polyketide\_synthase | BGC0000058 | FD-891\_biosynthetic\_gene\_cluster | 31 | 40.7865665046 | 357 | 1.1e-97 |
| CAF05649.1 | TubD\_protein | BGC0001053 | Tubulysin\_biosynthetic\_gene\_cluster | 30 | 40.6098099867 | 356 | 1.8e-97 |
| CCA29203.1 | non-ribosomal\_peptide\_synthetase/polyketide\_synthase | BGC0000955 | Althiomycin\_biosynthetic\_gene\_cluster | 30 | 40.0353513036 | 356 | 3.1e-97 |
| AHB82065.1 | polyketide\_synthase | BGC0001231 | Microsclerodermins\_biosynthetic\_gene\_cluster | 34 | 28.6345558992 | 356 | 3.1e-97 |
| EWG54266.1 | hypothetical\_protein | BGC0001190 | Fusaric\_acid\_biosynthetic\_gene\_cluster | 28 | 42.0680512594 | 356 | 3.1e-97 |
| AJW65408.1 | type\_I\_modular\_polyketide\_synthase | BGC0001195 | Nocardiopsin\_biosynthetic\_gene\_cluster | 29 | 40.9191338931 | 354 | 9e-97 |
| AHD05614.1 | putative\_non-ribosomal\_peptide\_ligase/\_polyketide\_synthase\_hybrid | BGC0001033 | Paenilamicin\_biosynthetic\_gene\_cluster | 29 | 40.0353513036 | 354 | 1.2e-96 |
| AAC68815.1 | FK506\_polyketide\_synthase | BGC0000353 | FK506\_biosynthetic\_gene\_cluster | 30 | 40.6539991162 | 354 | 1.2e-96 |
| ADH04682.1 | polyketide\_synthase | BGC0001344 | Tubulysin\_biosynthetic\_gene\_cluster | 30 | 39.9911621741 | 353 | 1.5e-96 |
| AAO06917.1 | GdmAII | BGC0000066 | Geldanamycin\_biosynthetic\_gene\_cluster | 29 | 40.9633230225 | 353 | 2e-96 |
| ACY06290.1 | type\_I\_polyketide\_synthase | BGC0001042 | Sanglifehrin\_A\_biosynthetic\_gene\_cluster | 29 | 41.8912947415 | 353 | 2e-96 |
| AEZ53948.1 | polyketide\_synthase | BGC0000144 | Salinomycin\_biosynthetic\_gene\_cluster | 29 | 40.167918692 | 353 | 2e-96 |
| AJO72742.1 | Type\_I\_modular\_polyketide\_synthase | BGC0001381 | Brasilinolide\_biosynthetic\_gene\_cluster | 30 | 39.0631904551 | 353 | 2e-96 |
| BAB69195.1 | modular\_polyketide\_synthase | BGC0000117 | Oligomycin\_biosynthetic\_gene\_cluster | 30 | 41.7587273531 | 352 | 2.6e-96 |
| AHE80996.1 | PieA6 | BGC0001169 | Piericidin\_A1\_biosynthetic\_gene\_cluster | 29 | 41.6261599646 | 351 | 5.8e-96 |
| ABA02240.1 | polyketide\_synthase | BGC0000098 | Monacolin\_K\_biosynthetic\_gene\_cluster | 29 | 43.3495360141 | 351 | 5.8e-96 |
| ALP32045.1 | CycE | BGC0001293 | Cyclizidine\_biosynthetic\_gene\_cluster | 28 | 45.0729120636 | 351 | 7.6e-96 |
| ABB86409.1 | GelB | BGC0000067 | Geldanamycin\_biosynthetic\_gene\_cluster | 29 | 40.8749447636 | 351 | 1e-95 |
| ADF88262.1 | mixed\_nonribosomal\_peptide\_synthetase/\_polyketide\_synthase | BGC0000979 | Cylindrospermopsin\_biosynthetic\_gene\_cluster | 32 | 33.4511710119 | 351 | 1e-95 |
| ADF88265.1 | mixed\_nonribosomal\_peptide\_synthetase/\_polyketide\_synthase | BGC0000980 | Cylindrospermopsin\_biosynthetic\_gene\_cluster | 32 | 33.4511710119 | 351 | 1e-95 |
| ANC94966.1 | AlmHI | BGC0001396 | Aldgamycin\_biosynthetic\_gene\_cluster | 30 | 39.7702165267 | 351 | 1e-95 |
| AAU93806.2 | polyketide\_synthase\_modules\_3\_and\_4 | BGC0000054 | Erythromycin\_biosynthetic\_gene\_cluster | 29 | 38.8864339373 | 350 | 1.3e-95 |
| AMY15068.1 | hexaketide\_synthase\_MF-SQHKS | BGC0001339 | Squalestatin\_S1\_biosynthetic\_gene\_cluster | 28 | 41.847105612 | 349 | 2.2e-95 |
| ADH04680.1 | hybrid\_polyketide\_synthase/non-ribosomal\_peptide\_synthetase | BGC0001344 | Tubulysin\_biosynthetic\_gene\_cluster | 30 | 40.2121078215 | 349 | 3.8e-95 |
| BAC76492.1 | lankamycin\_synthase\_LkmAII | BGC0000085 | Lankamycin\_biosynthetic\_gene\_cluster | 28 | 44.9403446752 | 348 | 6.5e-95 |
| ADC79620.1 | BafAV | BGC0000028 | Bafilomycin\_biosynthetic\_gene\_cluster | 29 | 40.167918692 | 348 | 6.5e-95 |
| CAJ46689.1 | polyketide\_synthase | BGC0000969 | Chondramide\_biosynthetic\_gene\_cluster | 32 | 38.8864339373 | 348 | 6.5e-95 |
| AGC09485.1 | LobS2 | BGC0001183 | Lobophorin\_biosynthetic\_gene\_cluster | 28 | 42.3331860362 | 347 | 1.1e-94 |
| ABL74938.1 | TlmVIII | BGC0001048 | Tallysomycin\_biosynthetic\_gene\_cluster | 30 | 40.8749447636 | 346 | 1.9e-94 |
| AGI99482.1 | Type\_I\_polyketide\_synthase | BGC0001004 | Lobophorin\_biosynthetic\_gene\_cluster | 27 | 46.7962881131 | 346 | 2.5e-94 |
| CDG12864.1 | non-ribosomal\_peptide\_synthetase | BGC0001415 | Althiomycin\_biosynthetic\_gene\_cluster | 29 | 41.8912947415 | 346 | 2.5e-94 |
| CCM44338.1 | putative\_polyketide\_synthase | BGC0001056 | Zeamine\_biosynthetic\_gene\_cluster | 29 | 40.1237295625 | 346 | 3.2e-94 |
| AAP33839.2 | ochratoxin\_A\_polyketide\_synthase | BGC0001030 | Ochratoxin\_A\_biosynthetic\_gene\_cluster | 31 | 33.5395492709 | 346 | 3.2e-94 |
| ABB90283.1 | polyketide\_synthase | BGC0001057 | Zearalenone\_biosynthetic\_gene\_cluster | 27 | 52.5408749448 | 346 | 3.2e-94 |
| AAF86393.1 | FkbB | BGC0000994 | FK520\_biosynthetic\_gene\_cluster | 30 | 40.1237295625 | 344 | 1.2e-93 |
| AHB82054.1 | polyketide\_synthase | BGC0001019 | Microsclerodermins\_biosynthetic\_gene\_cluster | 33 | 28.8555015466 | 343 | 1.6e-93 |
| AGC09499.1 | LobS4 | BGC0001183 | Lobophorin\_biosynthetic\_gene\_cluster | 28 | 40.9191338931 | 343 | 2.1e-93 |
| ABB76806.1 | polyketide\_synthase\_2 | BGC0000155\_c2 | T-toxin\_biosynthetic\_gene\_cluster | 27 | 54.1758727353 | 343 | 2.1e-93 |
| AAD34559.1 | polyketide\_synthase | BGC0000089 | Lovastatin\_biosynthetic\_gene\_cluster | 29 | 42.9518338489 | 342 | 2.7e-93 |
| ABF87031.1 | non-ribosomal\_peptide\_synthetase/polyketide\_synthase | BGC0000393 | Myxoprincomide\_biosynthetic\_gene\_cluster | 27 | 41.4052143173 | 342 | 2.7e-93 |
| AAM81584.2 | putative\_type\_I\_polyketide\_synthase | BGC0000047 | Dihydrochalcomycin\_biosynthetic\_gene\_cluster | 29 | 39.7702165267 | 342 | 3.5e-93 |
| BAQ25466.1 | polyketide\_synthase | BGC0001264 | Betaenone\_A\_/\_betaenone\_B\_/\_betaenone\_C\_biosynthetic\_gene\_cluster | 28 | 46.133451171 | 342 | 4.6e-93 |
| EHA22196.1 | polyketide\_synthase | BGC0000170 | Yanuthone\_D\_biosynthetic\_gene\_cluster | 30 | 39.9911621741 | 341 | 7.9e-93 |
| ABA02239.1 | polyketide\_synthase | BGC0000098 | Monacolin\_K\_biosynthetic\_gene\_cluster | 28 | 47.5475033142 | 340 | 1.8e-92 |
| BAD97694.1 | Aft9-1 | BGC0000003 | AF-toxin\_biosynthetic\_gene\_cluster | 31 | 36.8979231109 | 339 | 3e-92 |
| AGY62753.1 | EbeA | BGC0000051 | Ebelactone\_biosynthetic\_gene\_cluster | 29 | 36.4118426867 | 339 | 3e-92 |
| ADN43685.1 | DmbS | BGC0001136 | Desmethylbassianin\_biosynthetic\_gene\_cluster | 28 | 48.8289880689 | 338 | 5.1e-92 |
| CCC55921.1 | non-ribosomal\_peptide\_synthetase/polyketide\_synthase\_hybrid\_protein | BGC0000973 | Collismycin\_A\_biosynthetic\_gene\_cluster | 29 | 41.1400795404 | 338 | 5.1e-92 |
| ACS68554.1 | hybrid\_PKS-NRPS\_protein | BGC0001026 | NG-391\_biosynthetic\_gene\_cluster | 28 | 46.133451171 | 337 | 1.1e-91 |
| AHA38200.1 | GphG | BGC0000069 | Gephyronic\_acid\_biosynthetic\_gene\_cluster | 31 | 36.6327883341 | 336 | 1.9e-91 |
| AAM54078.1 | polyketide\_synthase | BGC0000020 | Ansamitocin\_biosynthetic\_gene\_cluster | 29 | 43.5704816615 | 336 | 2.5e-91 |
| EJK79843.1 | amino\_acid\_adenylation\_enzyme/thioester\_reductase\_family\_protein | BGC0000436 | Syringolin\_A\_biosynthetic\_gene\_cluster | 29 | 40.3888643394 | 336 | 3.3e-91 |
| CAL69597.1 | nonribosomal\_peptide\_synthase | BGC0001049 | Tenellin\_biosynthetic\_gene\_cluster | 28 | 49.0941228458 | 334 | 7.4e-91 |
| WP\_063764078.1 | polyketide\_synthase | BGC0001348 | JBIR-100\_biosynthetic\_gene\_cluster | 29 | 41.4494034468 | 334 | 9.7e-91 |
| BAQ25482.1 | type\_I\_polyketide\_synthase | BGC0001288 | Maklamicin\_biosynthetic\_gene\_cluster | 33 | 30.9323906319 | 334 | 9.7e-91 |
| AHB82057.1 | polyketide\_synthase | BGC0001019 | Microsclerodermins\_biosynthetic\_gene\_cluster | 29 | 40.3446752099 | 333 | 1.6e-90 |
| ABX37384.1 | Beta-ketoacyl\_synthase | BGC0000984 | Delftibactin\_biosynthetic\_gene\_cluster | 29 | 41.7145382236 | 333 | 2.2e-90 |
| AJO72737.1 | Type\_I\_modular\_polyketide\_synthase | BGC0001381 | Brasilinolide\_biosynthetic\_gene\_cluster | 28 | 52.09898365 | 333 | 2.2e-90 |
| CAJ87591.1 | putative\_peptide/polyketide\_synthetase | BGC0001055 | Yersiniabactin\_biosynthetic\_gene\_cluster | 28 | 40.8307556341 | 332 | 3.7e-90 |
| CAJ76298.1 | putative\_hybrid\_polyketide-non-ribosomal\_peptide\_synthetase | BGC0000972 | Colibactin\_biosynthetic\_gene\_cluster | 28 | 43.526292532 | 332 | 4.8e-90 |
| CBF80487.1 | hybrid\_PKS-NRPS\_(Eurofung) | BGC0000959 | Aspyridone\_biosynthetic\_gene\_cluster | 28 | 46.7079098542 | 330 | 1.4e-89 |
| CAD70195.1 | non-ribosomal\_peptide\_synthetase | BGC0001047 | Syringolin\_A\_biosynthetic\_gene\_cluster | 28 | 42.2448077773 | 330 | 1.8e-89 |
| AAV66110.2 | fusaridione\_A\_synthetase | BGC0000992 | Fusaridione\_A\_biosynthetic\_gene\_cluster | 30 | 37.9584622183 | 330 | 1.8e-89 |
| ABC36203.1 | thiotemplate\_mechanism\_natural\_product\_synthetase | BGC0001102 | Malleilactone\_biosynthetic\_gene\_cluster | 29 | 41.935483871 | 328 | 6.9e-89 |
| CAA73127.1 | HMWP1\_protein | BGC0000467 | Yersiniabactin\_biosynthetic\_gene\_cluster | 28 | 40.8307556341 | 328 | 6.9e-89 |
| ABO15888.1 | polyketide\_synthase | BGC0000132 | Pyrrolomycin\_biosynthetic\_gene\_cluster | 31 | 33.8488731772 | 327 | 9e-89 |
| AKC54422.1 | fumosorinone\_biosynthesis\_polyketide\_synthase | BGC0001218 | Fumosorinone\_biosynthetic\_gene\_cluster | 28 | 48.1219619973 | 327 | 1.5e-88 |
| BAN19720.1 | polyketide\_synthase | BGC0001252 | ACR-toxin\_biosynthetic\_gene\_cluster | 27 | 45.4264250994 | 326 | 2e-88 |
| BAC20564.1 | polyketide\_synthase | BGC0000039 | Compactin\_biosynthetic\_gene\_cluster | 27 | 47.1056120194 | 326 | 2e-88 |
| AJO72734.1 | Type\_I\_modular\_polyketide\_synthase | BGC0001381 | Brasilinolide\_biosynthetic\_gene\_cluster | 30 | 41.1842686699 | 326 | 2.6e-88 |
| AGO86662.1 | equisetin\_synthetase | BGC0001255 | Equisetin\_biosynthetic\_gene\_cluster | 27 | 45.9566946531 | 324 | 1e-87 |
| ACF35447.1 | MbcAIII | BGC0000090 | Macbecin\_biosynthetic\_gene\_cluster | 26 | 51.8780380027 | 323 | 2.2e-87 |
| ACZ57548.1 | polyketide\_synthase | BGC0000046 | Depudecin\_biosynthetic\_gene\_cluster | 27 | 54.2200618648 | 322 | 5e-87 |
| BAQ25511.1 | type\_I\_polyketide\_synthase | BGC0001288 | Maklamicin\_biosynthetic\_gene\_cluster | 34 | 29.2090145824 | 322 | 5e-87 |
| EAW09117.1 | hybrid\_NRPS/PKS\_enzyme,\_putative | BGC0000983 | Cytochalasin\_biosynthetic\_gene\_cluster | 27 | 48.6080424216 | 321 | 1.1e-86 |
| EAA65604.1 | hypothetical\_protein | BGC0000022 | Asperfuranone\_biosynthetic\_gene\_cluster | 26 | 57.6226248343 | 320 | 1.9e-86 |
| AKA54627.1 | PKS | BGC0001216 | Splenocin\_biosynthetic\_gene\_cluster | 28 | 40.4772425983 | 318 | 7.2e-86 |
| AFV52200.1 | polyketide\_synthase\_module | BGC0000081 | Kedarcidin\_biosynthetic\_gene\_cluster | 29 | 40.6539991162 | 318 | 7.2e-86 |
| CBJ89766.1 | Polyketide\_synthase\_involved\_in\_xenocoumacin\_synthesis | BGC0001054 | Xenocoumacin\_biosynthetic\_gene\_cluster | 27 | 41.5819708352 | 318 | 7.2e-86 |
| AEF33079.1 | polyketide\_synthase | BGC0001039 | Pyridomycin\_biosynthetic\_gene\_cluster | 29 | 40.3004860804 | 317 | 9.4e-86 |
| AAM54075.1 | polyketide\_synthase | BGC0000020 | Ansamitocin\_biosynthetic\_gene\_cluster | 28 | 43.526292532 | 317 | 1.2e-85 |
| WP\_010639241.1 | beta-ketoacyl\_synthase | BGC0000958 | Antimycin\_biosynthetic\_gene\_cluster | 28 | 40.4772425983 | 316 | 3.6e-85 |
| ADI59531.1 | CorI | BGC0001091 | Corallopyronin\_biosynthetic\_gene\_cluster | 32 | 26.469288555 | 315 | 6.1e-85 |
| EAU38971.1 | hypothetical\_protein | BGC0001122 | Isoflavipucine\_biosynthetic\_gene\_cluster | 28 | 46.7962881131 | 312 | 3.9e-84 |
| CCA89328.1 | mixed\_trans-AT\_type\_I\_polyketide\_synthase/nonribosomal\_peptide\_synthetase | BGC0001111 | Rhizopodins\_biosynthetic\_gene\_cluster | 34 | 26.8669907203 | 311 | 1.1e-83 |
| AAY89052.1 | polyketide\_synthase | BGC0001069 | Chivosazole\_biosynthetic\_gene\_cluster | 34 | 24.4365885992 | 310 | 1.5e-83 |
| AAG02357.1 | polyketide\_synthase | BGC0000963 | Bleomycin\_biosynthetic\_gene\_cluster | 29 | 40.3446752099 | 309 | 3.3e-83 |
| ADN68477.1 | SorB | BGC0000184 | Sorangicin\_biosynthetic\_gene\_cluster | 36 | 22.9341581971 | 309 | 3.3e-83 |
| CAQ34917.1 | polyketide\_synthase | BGC0000986 | Dkxanthene\_biosynthetic\_gene\_cluster | 33 | 30.7998232435 | 308 | 7.4e-83 |
| AIU36103.1 | LglD | BGC0000180 | Legioliulin\_biosynthetic\_gene\_cluster | 35 | 23.1992929739 | 308 | 7.4e-83 |
| ADN68483.1 | SorH | BGC0000184 | Sorangicin\_biosynthetic\_gene\_cluster | 36 | 23.022536456 | 307 | 1.3e-82 |
| CAN93348.1 | polyketide\_synthase | BGC0000179 | Etnangien\_biosynthetic\_gene\_cluster | 34 | 24.5691559876 | 307 | 1.7e-82 |
| ADN68480.1 | SorE | BGC0000184 | Sorangicin\_biosynthetic\_gene\_cluster | 35 | 24.5249668582 | 305 | 3.7e-82 |
| AKQ22696.1 | malonyl\_CoA-acyl\_carrier\_protein\_transacylase | BGC0001186 | Misakinolide\_biosynthetic\_gene\_cluster | 34 | 27.2205037561 | 305 | 3.7e-82 |
| ADI59534.1 | CorL | BGC0001091 | Corallopyronin\_biosynthetic\_gene\_cluster | 33 | 24.3923994697 | 305 | 4.8e-82 |
| EAQ86385.1 | hypothetical\_protein | BGC0001405 | Chaetoviridin\_/\_chaetomugilin\_biosynthetic\_gene\_cluster | 30 | 34.3791427309 | 305 | 6.3e-82 |
| CAG23977.1 | polyketide\_synthase\_type\_I | BGC0000176 | Difficidin\_biosynthetic\_gene\_cluster | 35 | 24.3040212108 | 304 | 1.1e-81 |
| AHB38498.1 | EpxE | BGC0000346 | Epoxomicin\_biosynthetic\_gene\_cluster | 29 | 38.5329209015 | 304 | 1.4e-81 |
| ADD82940.1 | Bat2 | BGC0001099 | Kalimantacin\_/\_batumin\_biosynthetic\_gene\_cluster | 34 | 23.5528060097 | 303 | 2.4e-81 |
| ADN68476.1 | SorA | BGC0000184 | Sorangicin\_biosynthetic\_gene\_cluster | 34 | 24.1714538224 | 303 | 2.4e-81 |
| CBJ89760.1 | Polyketide\_synthase\_involved\_in\_xenocoumacin\_synthesis | BGC0001054 | Xenocoumacin\_biosynthetic\_gene\_cluster | 30 | 37.2514361467 | 302 | 3.1e-81 |
| EAU29808.1 | hypothetical\_protein | BGC0001400 | Citreoviridin\_biosynthetic\_gene\_cluster | 28 | 41.6703490941 | 301 | 6.9e-81 |
| ABF92489.1 | mixed\_type\_I\_polyketide\_synthase\_-\_peptide\_synthetase | BGC0001025 | Myxovirescin\_biosynthetic\_gene\_cluster | 35 | 24.1714538224 | 300 | 1.2e-80 |
| ABF85931.1 | non-ribosomal\_peptide\_synthase/polyketide\_synthase\_Ta1 | BGC0001025 | Myxovirescin\_biosynthetic\_gene\_cluster | 34 | 24.1714538224 | 300 | 1.2e-80 |
| AAY32965.1 | DszB | BGC0001093 | Disorazole\_biosynthetic\_gene\_cluster | 34 | 25.2319929297 | 298 | 5.9e-80 |
| CAL69894.1 | RhiF\_protein | BGC0001112 | Rhizoxins\_biosynthetic\_gene\_cluster | 36 | 24.5249668582 | 297 | 1.7e-79 |
| CAG23966.1 | polyketide\_synthase\_type\_I | BGC0000181 | Macrolactin\_biosynthetic\_gene\_cluster | 33 | 24.0388864339 | 296 | 2.9e-79 |
| CTQ34882.1 | AtcE;\_polyketide\_synthase,\_modules\_5-7 | BGC0001301 | Anthracimycin\_biosynthetic\_gene\_cluster | 34 | 24.3923994697 | 295 | 3.8e-79 |
| AIJ04683.1 | polyketide\_synthase | BGC0001383 | Macrolactin\_biosynthetic\_gene\_cluster | 33 | 23.7737516571 | 295 | 3.8e-79 |
| ALD83703.1 | tAT\_polyketide\_synthase | BGC0001299 | Chlorotonil\_biosynthetic\_gene\_cluster | 35 | 23.3760494918 | 295 | 5e-79 |
| AAM12913.2 | MmpD | BGC0000182 | Mupirocin\_biosynthetic\_gene\_cluster | 33 | 25.3203711887 | 294 | 8.5e-79 |
| ALD83687.1 | tAT\_polyketide\_synthase | BGC0001300 | Anthracimycin\_biosynthetic\_gene\_cluster | 35 | 22.4038886434 | 294 | 1.1e-78 |
| ABP57747.1 | DepC | BGC0000993 | FK228\_biosynthetic\_gene\_cluster | 35 | 22.7132125497 | 293 | 1.9e-78 |
| ACY01391.1 | AT-less\_polyketide\_synthase | BGC0000177 | Dorrigocin\_/\_migrastatin\_biosynthetic\_gene\_cluster | 33 | 25.4087494476 | 293 | 2.5e-78 |
| BAP05595.1 | CalG | BGC0000967 | Calyculin\_biosynthetic\_gene\_cluster | 35 | 23.862129916 | 292 | 4.2e-78 |
| AKQ22699.1 | malonyl\_CoA-acyl\_carrier\_protein\_transacylase | BGC0001186 | Misakinolide\_biosynthetic\_gene\_cluster | 33 | 24.3482103403 | 290 | 1.2e-77 |
| ABI91469.1 | beta-ketoacyl\_synthase | BGC0001094 | Enacyloxin\_biosynthetic\_gene\_cluster | 36 | 21.6526734423 | 290 | 2.1e-77 |
| AEC04361.1 | polyketide\_synthase | BGC0000178 | Elansolid\_biosynthetic\_gene\_cluster | 35 | 22.4922669023 | 290 | 2.1e-77 |
| ALD83702.1 | tAT\_polyketide\_synthase | BGC0001299 | Chlorotonil\_biosynthetic\_gene\_cluster | 33 | 25.1878038003 | 289 | 2.7e-77 |
| CAJ57411.1 | polyketide\_synthase\_type\_I | BGC0000176 | Difficidin\_biosynthetic\_gene\_cluster | 33 | 25.9390190013 | 289 | 2.7e-77 |
| AIC32694.1 | FR9GH | BGC0001113 | Spliceostatin\_/\_FR901464\_biosynthetic\_gene\_cluster | 34 | 24.6575342466 | 289 | 3.6e-77 |
| ADH01489.1 | type\_I\_polyketide\_synthase | BGC0000995 | FR901464\_biosynthetic\_gene\_cluster | 34 | 24.6575342466 | 289 | 3.6e-77 |
| CAN93347.1 | Polyketide\_synthase | BGC0000179 | Etnangien\_biosynthetic\_gene\_cluster | 34 | 23.3760494918 | 289 | 4.7e-77 |
| AFX60313.1 | polyketide\_synthase | BGC0001031 | Oocydin\_A\_biosynthetic\_gene\_cluster | 34 | 24.2156429518 | 289 | 4.7e-77 |
| CBX87032.1 | BcBOA9\_protein | BGC0001243\_c3 | Botryenalol\_/\_botrydial\_/\_dihydrobotrydial\_/\_botryendial\_/ | 28 | 41.935483871 | 288 | 6.1e-77 |
| CTQ34881.1 | AtcD;\_polyketide\_synthase,\_modules\_1-4 | BGC0001301 | Anthracimycin\_biosynthetic\_gene\_cluster | 34 | 24.1714538224 | 288 | 7.9e-77 |
| ADI59532.1 | CorJ | BGC0001091 | Corallopyronin\_biosynthetic\_gene\_cluster | 35 | 24.2156429518 | 288 | 7.9e-77 |
| AFN27481.1 | pks\_BonB | BGC0000173 | Bongkrekic\_acid\_biosynthetic\_gene\_cluster | 34 | 23.7295625276 | 288 | 7.9e-77 |
| BAF50727.1 | hybrid\_polyketide\_synthase-non\_ribosomal\_peptide\_synthetase | BGC0001116 | Virginiamycin\_biosynthetic\_gene\_cluster | 32 | 27.1321254971 | 288 | 7.9e-77 |
| ABS90470.1 | NRPS/PKS | BGC0001106 | Oxazolomycin\_biosynthetic\_gene\_cluster | 36 | 22.1387538665 | 287 | 1e-76 |
| ALD83686.1 | tAT\_polyketide\_synthase | BGC0001300 | Anthracimycin\_biosynthetic\_gene\_cluster | 35 | 24.2156429518 | 287 | 1e-76 |
| CBK62733.1 |  | BGC0001115 | Thiomarinol\_biosynthetic\_gene\_cluster | 31 | 29.1648254529 | 287 | 1e-76 |
| AMH40443.1 | PKS | BGC0001350 | Phormidolide\_biosynthetic\_gene\_cluster | 36 | 21.7852408308 | 287 | 1.4e-76 |
| CAG23958.2 | polyketide\_synthase | BGC0001089 | Bacillaene\_biosynthetic\_gene\_cluster | 32 | 23.3318603624 | 287 | 1.8e-76 |
| ADA82585.1 | hybrid\_trans-AT\_polyketide\_synthase\_-\_nonribosomal\_peptide\_synthetase | BGC0001110 | Psymberin\_biosynthetic\_gene\_cluster | 34 | 22.7574016792 | 287 | 1.8e-76 |
| AFN27480.1 | pks\_BonA | BGC0000173 | Bongkrekic\_acid\_biosynthetic\_gene\_cluster | 33 | 24.4365885992 | 287 | 1.8e-76 |
| AAS47562.1 | mixed\_type\_I\_polyketide\_synthase\_-\_peptide\_synthetase | BGC0001108 | Pederin\_biosynthetic\_gene\_cluster | 34 | 23.5528060097 | 286 | 2.3e-76 |
| ctg1\_orf8 |  | BGC0001109 | Pederin\_biosynthetic\_gene\_cluster | 34 | 23.5528060097 | 286 | 2.3e-76 |
| AAY32964.1 | DszA | BGC0001093 | Disorazole\_biosynthetic\_gene\_cluster | 34 | 24.1272646929 | 285 | 3.9e-76 |
| ADN68482.1 | SorG | BGC0000184 | Sorangicin\_biosynthetic\_gene\_cluster | 34 | 23.950508175 | 285 | 3.9e-76 |
| AGN11882.1 | TstGH | BGC0001114 | Thailanstatin\_biosynthetic\_gene\_cluster | 35 | 23.6853733981 | 285 | 3.9e-76 |
| BAP05593.1 | CalE | BGC0000967 | Calyculin\_biosynthetic\_gene\_cluster | 33 | 26.7344233319 | 285 | 3.9e-76 |
| ABI91467.1 | beta-ketoacyl\_synthase | BGC0001094 | Enacyloxin\_biosynthetic\_gene\_cluster | 33 | 23.6411842687 | 285 | 5.1e-76 |
| AAY89049.1 | polyketide\_synthase | BGC0001069 | Chivosazole\_biosynthetic\_gene\_cluster | 34 | 23.2434821034 | 285 | 5.1e-76 |
| AEZ54376.1 | PieA3 | BGC0000124 | Piericidin\_A1\_biosynthetic\_gene\_cluster | 32 | 29.6067167477 | 285 | 6.7e-76 |
| ABC34675.1 | polyketide\_synthase,\_putative | BGC0000186 | Thailandamide\_biosynthetic\_gene\_cluster | 33 | 24.1272646929 | 285 | 6.7e-76 |
| CAG23967.1 | polyketide\_synthase\_type\_I | BGC0000181 | Macrolactin\_biosynthetic\_gene\_cluster | 33 | 24.1714538224 | 284 | 8.8e-76 |
| CAG23983.1 | polyketide\_synthase\_type\_I | BGC0000176 | Difficidin\_biosynthetic\_gene\_cluster | 34 | 22.5806451613 | 284 | 8.8e-76 |
| BAQ25481.1 | type\_I\_polyketide\_synthase | BGC0001288 | Maklamicin\_biosynthetic\_gene\_cluster | 34 | 24.5691559876 | 284 | 1.1e-75 |
| AHE80993.1 | PieA3 | BGC0001169 | Piericidin\_A1\_biosynthetic\_gene\_cluster | 31 | 29.6509058772 | 284 | 1.1e-75 |
| AFX60311.1 | polyketide\_synthase | BGC0001031 | Oocydin\_A\_biosynthetic\_gene\_cluster | 36 | 21.8736190897 | 283 | 2e-75 |
| CCA89326.1 | mixed\_trans-AT\_type\_I\_polyketide\_synthase/nonribosomal\_peptide\_synthetase | BGC0001111 | Rhizopodins\_biosynthetic\_gene\_cluster | 32 | 24.5249668582 | 283 | 2e-75 |
| ABS90475.1 | PKS | BGC0001106 | Oxazolomycin\_biosynthetic\_gene\_cluster | 33 | 24.0830755634 | 283 | 2e-75 |
| AFX60309.1 | polyketide\_synthase | BGC0001031 | Oocydin\_A\_biosynthetic\_gene\_cluster | 34 | 24.4365885992 | 283 | 2.6e-75 |
| AIC32693.1 | FR9DEF | BGC0001113 | Spliceostatin\_/\_FR901464\_biosynthetic\_gene\_cluster | 36 | 23.5086168802 | 282 | 3.3e-75 |
| ADH01487.1 | polyketide\_synthase | BGC0001096 | FR901464\_biosynthetic\_gene\_cluster | 36 | 23.5086168802 | 282 | 3.3e-75 |
| AIJ04684.1 | polyketide\_synthase | BGC0001383 | Macrolactin\_biosynthetic\_gene\_cluster | 33 | 24.0830755634 | 282 | 3.3e-75 |
| CAG23959.2 | polyketide\_synthase | BGC0001089 | Bacillaene\_biosynthetic\_gene\_cluster | 34 | 24.3923994697 | 282 | 3.3e-75 |
| AAM12909.2 | MmpA | BGC0000182 | Mupirocin\_biosynthetic\_gene\_cluster | 33 | 24.3040212108 | 282 | 4.4e-75 |
| CBK62731.1 |  | BGC0001115 | Thiomarinol\_biosynthetic\_gene\_cluster | 33 | 23.1551038445 | 282 | 4.4e-75 |
| AFX60336.1 | polyketide\_synthase | BGC0001032 | Oocydin\_A\_biosynthetic\_gene\_cluster | 34 | 23.1551038445 | 282 | 5.7e-75 |
| AFX60334.1 | polyketide\_synthase | BGC0001032 | Oocydin\_A\_biosynthetic\_gene\_cluster | 36 | 21.8736190897 | 281 | 7.4e-75 |
| ABC35522.1 | thiotemplate\_mechanism\_natural\_product\_synthetase | BGC0000186 | Thailandamide\_biosynthetic\_gene\_cluster | 33 | 24.790101635 | 281 | 9.7e-75 |
| CCA89327.1 | trans-AT\_type\_I\_polyketide\_synthase | BGC0001111 | Rhizopodins\_biosynthetic\_gene\_cluster | 31 | 25.8506407424 | 281 | 9.7e-75 |
| AFX60341.1 | polyketide\_synthase | BGC0001032 | Oocydin\_A\_biosynthetic\_gene\_cluster | 32 | 26.1157755192 | 280 | 1.3e-74 |
| AAN85523.1 | polyketide\_synthase | BGC0001101 | Leinamycin\_biosynthetic\_gene\_cluster | 36 | 24.1272646929 | 280 | 1.3e-74 |
| AMH40422.1 | PKS | BGC0001350 | Phormidolide\_biosynthetic\_gene\_cluster | 34 | 24.5691559876 | 280 | 1.3e-74 |
| AAV97870.1 | OnnB | BGC0001105 | Onnamide\_biosynthetic\_gene\_cluster | 30 | 25.0994255413 | 280 | 1.7e-74 |
| CAJ57409.1 | polyketide\_synthase\_type\_I | BGC0000176 | Difficidin\_biosynthetic\_gene\_cluster | 34 | 23.7295625276 | 280 | 1.7e-74 |
| CBW45749.1 | hybrid\_PKS/NRPS\_(PKSI) | BGC0000952\_c3 | Pristinamycin\_biosynthetic\_gene\_cluster | 38 | 20.4153778171 | 280 | 2.2e-74 |
| ADN68478.1 | SorC | BGC0000184 | Sorangicin\_biosynthetic\_gene\_cluster | 34 | 23.7295625276 | 280 | 2.2e-74 |
| ABC34832.1 | polyketide\_synthase | BGC0000186 | Thailandamide\_biosynthetic\_gene\_cluster | 32 | 25.4971277066 | 279 | 2.8e-74 |
| AKA59448.1 | polyketide\_synthase | BGC0001203 | Clarexpoxcin\_biosynthetic\_gene\_cluster | 29 | 36.5885992046 | 279 | 3.7e-74 |
| ABK32260.1 | AmbF | BGC0000014 | Ambruticin\_biosynthetic\_gene\_cluster | 35 | 23.5969951392 | 279 | 3.7e-74 |
| ABC84465.1 | NigAX | BGC0000114 | Nigericin\_biosynthetic\_gene\_cluster | 31 | 29.916040654 | 279 | 3.7e-74 |
| AMH40421.1 | PKS | BGC0001350 | Phormidolide\_biosynthetic\_gene\_cluster | 33 | 24.7459125055 | 279 | 3.7e-74 |
| AIJ04681.1 | polyketide\_synthase | BGC0001383 | Macrolactin\_biosynthetic\_gene\_cluster | 33 | 23.9063190455 | 279 | 4.8e-74 |
| AFX60318.1 | polyketide\_synthase | BGC0001031 | Oocydin\_A\_biosynthetic\_gene\_cluster | 32 | 24.9668581529 | 279 | 4.8e-74 |
| AKQ22698.1 | malonyl\_CoA-acyl\_carrier\_protein\_transacylase | BGC0001186 | Misakinolide\_biosynthetic\_gene\_cluster | 33 | 23.5528060097 | 279 | 4.8e-74 |
| AFX60332.1 | polyketide\_synthase | BGC0001032 | Oocydin\_A\_biosynthetic\_gene\_cluster | 32 | 26.2041537782 | 278 | 6.3e-74 |
| AAC38075.1 | polyketide\_synthase\_type\_I | BGC0000127 | Pyoluteorin\_biosynthetic\_gene\_cluster | 34 | 25.629695095 | 278 | 8.2e-74 |
| AAO65796.1 | monensin\_polyketide\_synthase\_loading\_module\_and\_module\_1 | BGC0000100 | Monensin\_biosynthetic\_gene\_cluster | 34 | 26.469288555 | 277 | 1.1e-73 |
| AFX60317.1 | polyketide\_synthase | BGC0001031 | Oocydin\_A\_biosynthetic\_gene\_cluster | 34 | 23.4644277508 | 277 | 1.1e-73 |
| ADN68479.1 | SorD | BGC0000184 | Sorangicin\_biosynthetic\_gene\_cluster | 34 | 23.2876712329 | 277 | 1.4e-73 |
| CBK62724.1 |  | BGC0001115 | Thiomarinol\_biosynthetic\_gene\_cluster | 33 | 22.7574016792 | 277 | 1.4e-73 |
| CCC21123.1 | type-I\_polyketide\_synthases | BGC0000171 | 9-methylstreptimidone\_biosynthetic\_gene\_cluster | 34 | 25.1436146708 | 277 | 1.4e-73 |
| AEC04357.1 | polyketide\_synthase | BGC0000178 | Elansolid\_biosynthetic\_gene\_cluster | 32 | 24.4365885992 | 277 | 1.4e-73 |
| CAG23964.1 | polyketide\_synthase\_type\_I | BGC0000181 | Macrolactin\_biosynthetic\_gene\_cluster | 32 | 23.9063190455 | 276 | 2.4e-73 |
| ABM63529.1 | BryX | BGC0000174 | Bryostatin\_biosynthetic\_gene\_cluster | 33 | 23.6853733981 | 276 | 3.1e-73 |
| BAP05591.1 | CalC | BGC0000967 | Calyculin\_biosynthetic\_gene\_cluster | 34 | 22.7574016792 | 276 | 3.1e-73 |
| AAO65790.1 | ketosynthase-like\_protein | BGC0000100 | Monensin\_biosynthetic\_gene\_cluster | 31 | 30.4463102077 | 276 | 3.1e-73 |
| CAL69893.1 | RhiE\_protein | BGC0001112 | Rhizoxins\_biosynthetic\_gene\_cluster | 31 | 26.2925320371 | 276 | 3.1e-73 |
| AMH40423.1 | PKS | BGC0001350 | Phormidolide\_biosynthetic\_gene\_cluster | 37 | 23.1551038445 | 275 | 4.1e-73 |
| AFN27482.1 | pks\_BonC | BGC0000173 | Bongkrekic\_acid\_biosynthetic\_gene\_cluster | 33 | 23.2876712329 | 275 | 4.1e-73 |
| ADI59533.1 | CorK | BGC0001091 | Corallopyronin\_biosynthetic\_gene\_cluster | 35 | 22.0945647371 | 275 | 4.1e-73 |
| AEC04362.1 | polyketide\_synthase | BGC0000178 | Elansolid\_biosynthetic\_gene\_cluster | 31 | 27.7065841803 | 275 | 5.3e-73 |
| AAM12911.1 | MmpB | BGC0000182 | Mupirocin\_biosynthetic\_gene\_cluster | 34 | 22.8457799381 | 275 | 7e-73 |
| CAJ57410.1 | polyketide\_synthase\_type\_I | BGC0000176 | Difficidin\_biosynthetic\_gene\_cluster | 33 | 24.790101635 | 274 | 9.1e-73 |
| ACY01400.1 | AT-less\_polyketide\_synthase | BGC0000083 | Lactimidomycin\_biosynthetic\_gene\_cluster | 34 | 23.4202386213 | 274 | 1.2e-72 |
| CBW45748.1 | hybrid\_PKS/NRPS\_(PKSI) | BGC0000952\_c3 | Pristinamycin\_biosynthetic\_gene\_cluster | 33 | 23.4644277508 | 274 | 1.2e-72 |
| AIJ04686.1 | polyketide\_synthase | BGC0001383 | Macrolactin\_biosynthetic\_gene\_cluster | 34 | 22.7132125497 | 274 | 1.2e-72 |
| ABC33986.1 | polyketide\_synthase,\_putative | BGC0000186 | Thailandamide\_biosynthetic\_gene\_cluster | 33 | 22.8015908087 | 274 | 1.6e-72 |
| CAG23968.1 | polyketide\_synthase\_type\_I | BGC0000181 | Macrolactin\_biosynthetic\_gene\_cluster | 33 | 22.7132125497 | 273 | 2.6e-72 |
| AGN74892.1 | nonribosomal\_peptide\_synthetase/polyketide\_synthase\_hybrid\_protein | BGC0000459 | Griseoviridin\_/\_viridogrisein\_biosynthetic\_gene\_cluster | 37 | 20.3269995581 | 273 | 2.6e-72 |
| ABI91464.1 | beta-ketoacyl\_synthase | BGC0001094 | Enacyloxin\_biosynthetic\_gene\_cluster | 32 | 25.8948298719 | 272 | 3.5e-72 |
| CTQ34883.1 | AtcF;\_polyketide\_synthase,\_modules\_8-10 | BGC0001301 | Anthracimycin\_biosynthetic\_gene\_cluster | 33 | 23.8179407866 | 272 | 5.9e-72 |
| CAN93351.1 | polyketide\_synthase | BGC0000179 | Etnangien\_biosynthetic\_gene\_cluster | 33 | 24.5249668582 | 272 | 5.9e-72 |
| AIJ04680.1 | polyketide\_synthase | BGC0001383 | Macrolactin\_biosynthetic\_gene\_cluster | 31 | 24.2598320813 | 272 | 5.9e-72 |
| BAC76471.1 | type\_I\_polyketide\_synthase\_LkcF | BGC0001100 | Lankacidin\_biosynthetic\_gene\_cluster | 32 | 24.1272646929 | 271 | 1e-71 |
| ACY01401.1 | AT-less\_polyketide\_synthase | BGC0000083 | Lactimidomycin\_biosynthetic\_gene\_cluster | 35 | 22.4922669023 | 271 | 1e-71 |
| CAN89631.1 | putative\_polyketide\_synthase | BGC0001070 | Kirromycin\_biosynthetic\_gene\_cluster | 34 | 23.5969951392 | 271 | 1e-71 |
| ABM63527.1 | BryB | BGC0000174 | Bryostatin\_biosynthetic\_gene\_cluster | 32 | 24.5691559876 | 270 | 1.7e-71 |
| CAJ57408.1 | polyketide\_synthase\_type\_I | BGC0000176 | Difficidin\_biosynthetic\_gene\_cluster | 35 | 21.6968625718 | 270 | 2.2e-71 |
| AAS47564.1 | mixed\_type\_I\_polyketide\_synthase/nonribosomal\_peptide\_synthetase | BGC0001108 | Pederin\_biosynthetic\_gene\_cluster | 32 | 23.950508175 | 270 | 2.2e-71 |
| ctg1\_orf6 |  | BGC0001109 | Pederin\_biosynthetic\_gene\_cluster | 32 | 23.950508175 | 270 | 2.2e-71 |
| ALD83688.1 | tAT\_polyketide\_synthase | BGC0001300 | Anthracimycin\_biosynthetic\_gene\_cluster | 33 | 24.3482103403 | 269 | 2.9e-71 |
| CAG23965.1 | polyketide\_synthase\_type\_I | BGC0000181 | Macrolactin\_biosynthetic\_gene\_cluster | 31 | 24.2598320813 | 269 | 5e-71 |
| CAL69890.1 | RhiC\_protein | BGC0001112 | Rhizoxins\_biosynthetic\_gene\_cluster | 34 | 22.0945647371 | 269 | 5e-71 |
| AFN27483.1 | pks\_BonD | BGC0000173 | Bongkrekic\_acid\_biosynthetic\_gene\_cluster | 32 | 29.3857711003 | 268 | 6.5e-71 |
| CAE52339.1 | Polyketide\_non-ribosomal\_peptide\_synthase | BGC0001088 | Albicidin\_biosynthetic\_gene\_cluster | 31 | 25.8948298719 | 268 | 8.5e-71 |
| AFO59866.1 | ChxE | BGC0000175 | Cycloheximide\_/\_actiphenol\_biosynthetic\_gene\_cluster | 33 | 24.3482103403 | 267 | 1.5e-70 |
| AGN11881.1 | TstDEF | BGC0001114 | Thailanstatin\_biosynthetic\_gene\_cluster | 35 | 23.3318603624 | 267 | 1.5e-70 |
| CAN93350.1 | polyketide\_synthase | BGC0000179 | Etnangien\_biosynthetic\_gene\_cluster | 33 | 25.0552364118 | 267 | 1.5e-70 |
| ABF89568.1 | polyketide\_synthase | BGC0001025 | Myxovirescin\_biosynthetic\_gene\_cluster | 30 | 28.7671232877 | 267 | 1.5e-70 |
| AFX60340.1 | polyketide\_synthase | BGC0001032 | Oocydin\_A\_biosynthetic\_gene\_cluster | 33 | 23.6853733981 | 267 | 1.9e-70 |
| AIJ04685.1 | polyketide\_synthase | BGC0001383 | Macrolactin\_biosynthetic\_gene\_cluster | 33 | 21.2107821476 | 266 | 2.5e-70 |
| AEC04356.1 | polyketide\_synthase | BGC0000178 | Elansolid\_biosynthetic\_gene\_cluster | 32 | 23.950508175 | 266 | 3.2e-70 |
| CAG23969.1 | polyketide\_synthase\_type\_I | BGC0000181 | Macrolactin\_biosynthetic\_gene\_cluster | 33 | 21.2107821476 | 265 | 5.5e-70 |
| ABM63528.1 | BryC | BGC0000174 | Bryostatin\_biosynthetic\_gene\_cluster | 33 | 24.3923994697 | 265 | 5.5e-70 |
| CBW45741.1 | hybrid\_PKS/NRPS\_(PKSI) | BGC0000952\_c3 | Pristinamycin\_biosynthetic\_gene\_cluster | 32 | 24.5691559876 | 265 | 7.2e-70 |
| CAL69891.1 | RhiD\_protein | BGC0001112 | Rhizoxins\_biosynthetic\_gene\_cluster | 32 | 26.3367211666 | 264 | 9.4e-70 |
| AEH42473.1 | polyketide\_synthase | BGC0000032 | Calcimycin\_biosynthetic\_gene\_cluster | 31 | 30.1369863014 | 264 | 1.6e-69 |
| AEC04363.1 | polyketide\_synthase | BGC0000178 | Elansolid\_biosynthetic\_gene\_cluster | 34 | 23.6853733981 | 263 | 2.1e-69 |
| ABC87511.1 | polyketide\_synthase | BGC0001011 | Meridamycin\_biosynthetic\_gene\_cluster | 34 | 20.7247017234 | 263 | 2.1e-69 |
| ERM18799.1 | polyketide\_synthase | BGC0000172 | Basiliskamides\_biosynthetic\_gene\_cluster | 32 | 24.5691559876 | 263 | 2.1e-69 |
| ctg1\_orf22 |  | BGC0001013 | Meridamycin\_biosynthetic\_gene\_cluster | 34 | 20.7247017234 | 263 | 2.1e-69 |
| BAD38875.1 | polyketide\_synthase | BGC0000111 | Neocarzilin\_biosynthetic\_gene\_cluster | 35 | 22.7574016792 | 263 | 2.7e-69 |
| ABC35796.1 | polyketide\_synthase,\_putative | BGC0001102 | Malleilactone\_biosynthetic\_gene\_cluster | 32 | 24.4807777287 | 263 | 2.7e-69 |
| BAK64638.1 | polyketide\_synthase | BGC0000135 | Reveromycin\_biosynthetic\_gene\_cluster | 37 | 20.6805125939 | 262 | 3.6e-69 |
| AEH59109.1 | keto-hydroxyglutarate-aldolase/polyketide\_synthase | BGC0000385 | Lysobactin\_biosynthetic\_gene\_cluster | 34 | 24.1714538224 | 262 | 4.7e-69 |
| CAN89635.1 | putative\_polyketide\_synthase | BGC0001070 | Kirromycin\_biosynthetic\_gene\_cluster | 34 | 21.1224038886 | 262 | 6.1e-69 |
| CCA89329.1 | trans-AT\_type\_I\_polyketide\_synthase | BGC0001111 | Rhizopodins\_biosynthetic\_gene\_cluster | 32 | 25.1878038003 | 261 | 8e-69 |
| BAP05594.1 | CalF | BGC0000967 | Calyculin\_biosynthetic\_gene\_cluster | 31 | 26.1599646487 | 261 | 8e-69 |
| EPE34340.1 | Thiolase-like\_protein | BGC0001035 | Pneumocandin\_biosynthetic\_gene\_cluster | 33 | 20.8130799823 | 260 | 1.4e-68 |
| AAX98184.1 | polyketide\_synthase\_type\_I | BGC0000052 | ECO-02301\_biosynthetic\_gene\_cluster | 34 | 20.5479452055 | 260 | 1.8e-68 |
| AFL48521.1 | beta-ketosynthase | BGC0000084 | Laidlomycin\_biosynthetic\_gene\_cluster | 30 | 29.5625276182 | 260 | 1.8e-68 |
| ANF07288.1 | hrPKS | BGC0001385 | Byssochlamic\_acid\_/\_agnestadrides\_biosynthetic\_gene\_cluster | 34 | 20.4153778171 | 259 | 5.2e-68 |
| ACR12418.1 | modular\_polyketide\_synthase,\_type\_I\_PKS | BGC0000185 | Tartrolon\_biosynthetic\_gene\_cluster | 31 | 23.9063190455 | 258 | 8.8e-68 |
| AGS06823.1 | type\_I\_polyketide\_synthase,\_PedI-like\_protein | BGC0001092 | Diaphorin\_biosynthetic\_gene\_cluster | 30 | 25.4087494476 | 257 | 1.1e-67 |
| ABS90471.1 | PKS\_type\_I | BGC0001106 | Oxazolomycin\_biosynthetic\_gene\_cluster | 31 | 22.6690234202 | 257 | 1.1e-67 |
| ACY01392.1 | AT-less\_polyketide\_synthase | BGC0000177 | Dorrigocin\_/\_migrastatin\_biosynthetic\_gene\_cluster | 34 | 20.503756076 | 257 | 1.1e-67 |
| CAG23960.2 | hybrid\_NRPS/PKS | BGC0001089 | Bacillaene\_biosynthetic\_gene\_cluster | 31 | 24.0388864339 | 257 | 2e-67 |
| AGN74893.1 | nonribosomal\_peptide\_synthetase/polyketide\_synthase\_hybrid\_protein | BGC0000459 | Griseoviridin\_/\_viridogrisein\_biosynthetic\_gene\_cluster | 35 | 21.431727795 | 256 | 2.6e-67 |
| ADA69239.2 | trans-AT\_hybrid\_polyketide\_synthase-NRPS | BGC0001071 | Nosperin\_biosynthetic\_gene\_cluster | 31 | 23.8179407866 | 255 | 7.5e-67 |
| AAB66504.1 | tylactone\_synthase\_starter\_module\_and\_modules\_1\_&\_2 | BGC0000166 | Tylactone\_biosynthetic\_gene\_cluster | 35 | 21.9178082192 | 253 | 1.7e-66 |
| ABM63537.1 | BryA | BGC0000174 | Bryostatin\_biosynthetic\_gene\_cluster | 33 | 21.7852408308 | 253 | 2.8e-66 |
| ACY01390.1 | AT-less\_polyketide\_synthase | BGC0000177 | Dorrigocin\_/\_migrastatin\_biosynthetic\_gene\_cluster | 32 | 24.6575342466 | 252 | 4.8e-66 |
| AGS06887.1 | mixed\_type\_I\_polyketide\_synthase/non-ribosomal\_peptide\_synthetase,\_PedF-like\_protein | BGC0001092\_c2 | Diaphorin\_biosynthetic\_gene\_cluster | 30 | 23.7737516571 | 251 | 8.2e-66 |
| CAL69889.1 | RhiB\_protein | BGC0001112 | Rhizoxins\_biosynthetic\_gene\_cluster | 30 | 24.0830755634 | 251 | 8.2e-66 |
| AGN74894.1 | nonribosomal\_peptide\_synthetase/polyketide\_synthase\_hybrid\_protein | BGC0000459 | Griseoviridin\_/\_viridogrisein\_biosynthetic\_gene\_cluster | 33 | 23.4202386213 | 251 | 1.1e-65 |
| CAN93352.1 | polyketide\_synthase | BGC0000179 | Etnangien\_biosynthetic\_gene\_cluster | 32 | 23.0667255855 | 250 | 1.8e-65 |
| CAP95405.1 |  | BGC0001404 | Sorbicillin\_biosynthetic\_gene\_cluster | 34 | 20.1060539107 | 250 | 1.8e-65 |
| AGN11883.1 | TstI | BGC0001114 | Thailanstatin\_biosynthetic\_gene\_cluster | 31 | 25.8064516129 | 250 | 2.4e-65 |
| ACR50796.1 | putative\_polyketide\_synthase | BGC0000163 | Tetronasin\_biosynthetic\_gene\_cluster | 32 | 23.4202386213 | 248 | 5.3e-65 |
| AKQ22697.1 | malonyl\_CoA-acyl\_carrier\_protein\_transacylase | BGC0001186 | Misakinolide\_biosynthetic\_gene\_cluster | 31 | 24.5249668582 | 247 | 1.2e-64 |
| BAD38874.1 | polyketide\_synthase | BGC0000111 | Neocarzilin\_biosynthetic\_gene\_cluster | 34 | 23.4644277508 | 247 | 1.6e-64 |
| BAC76474.1 | type\_I\_polyketide\_synthase\_LkcC | BGC0001100 | Lankacidin\_biosynthetic\_gene\_cluster | 31 | 24.3923994697 | 247 | 2e-64 |
| CAN93349.1 | polyketide\_synthase | BGC0000179 | Etnangien\_biosynthetic\_gene\_cluster | 32 | 23.110914715 | 246 | 3.5e-64 |
| BAF50721.1 | hybrid\_non\_ribosomal\_peptide\_synthetase-polyketide\_synthase | BGC0001116 | Virginiamycin\_biosynthetic\_gene\_cluster | 34 | 20.9898365002 | 246 | 3.5e-64 |
| BAP05597.1 | CalI | BGC0000967 | Calyculin\_biosynthetic\_gene\_cluster | 30 | 25.2319929297 | 245 | 5.9e-64 |
| BAE93740.1 | type\_I\_polyketide\_synthase-related\_protein | BGC0000164 | Tetronomycin\_biosynthetic\_gene\_cluster | 32 | 23.1992929739 | 245 | 5.9e-64 |
| CAG23961.2 | polyketide\_synthase | BGC0001089 | Bacillaene\_biosynthetic\_gene\_cluster | 31 | 23.6411842687 | 243 | 2.2e-63 |
| AGS06824.1 | mixed\_type\_I\_polyketide\_synthase/non-ribosomal\_peptide\_synthetase,\_PedH-like\_protein | BGC0001092 | Diaphorin\_biosynthetic\_gene\_cluster | 30 | 24.1272646929 | 243 | 2.9e-63 |
| AAY89051.1 | hybrid\_nonribosomal\_peptide\_synthetase/polyketide\_synthase | BGC0001069 | Chivosazole\_biosynthetic\_gene\_cluster | 32 | 22.6248342908 | 242 | 5e-63 |
| BAC76476.1 | multifunctional\_polyketide-peptide\_synthase\_LkcA | BGC0001100 | Lankacidin\_biosynthetic\_gene\_cluster | 33 | 23.2876712329 | 241 | 1.1e-62 |
| ALV82446.1 | putative\_type\_I\_polyketide\_synthase | BGC0001382 | Svaricin\_biosynthetic\_gene\_cluster | 32 | 25.8948298719 | 240 | 1.5e-62 |
| ABC38101.1 | polyketide\_synthase | BGC0000964 | Burkholdac\_biosynthetic\_gene\_cluster | 34 | 22.6690234202 | 240 | 2.5e-62 |
| ADA69237.1 | trans-AT\_polyketide\_synthase | BGC0001071 | Nosperin\_biosynthetic\_gene\_cluster | 31 | 21.0340256297 | 239 | 4.2e-62 |
| AFK83819.1 | PtzC | BGC0001107 | Patellazole\_biosynthetic\_gene\_cluster | 32 | 24.9668581529 | 238 | 5.5e-62 |
| ADH01484.1 | putative\_type-I\_PKS | BGC0001096 | FR901464\_biosynthetic\_gene\_cluster | 30 | 22.8457799381 | 237 | 2.1e-61 |
| AIC32692.1 | FR9C | BGC0001113 | Spliceostatin\_/\_FR901464\_biosynthetic\_gene\_cluster | 30 | 22.8457799381 | 237 | 2.1e-61 |
| AGN11880.1 | TstC | BGC0001114 | Thailanstatin\_biosynthetic\_gene\_cluster | 32 | 23.862129916 | 235 | 8e-61 |
| ABS90472.1 | PKS | BGC0001106 | Oxazolomycin\_biosynthetic\_gene\_cluster | 32 | 22.8899690676 | 233 | 1.8e-60 |
| AHD05679.1 | putative\_non-ribosomal\_peptide\_ligase/\_polyketide\_synthase\_hybrid | BGC0000402 | Paenilarvins\_biosynthetic\_gene\_cluster | 31 | 23.6411842687 | 232 | 6.8e-60 |
| BAB69698.1 | iturin\_A\_synthetase\_A | BGC0001098 | Iturin\_biosynthetic\_gene\_cluster | 31 | 24.4365885992 | 231 | 1.2e-59 |
| BAF50722.1 | polyketide\_synthase | BGC0001116 | Virginiamycin\_biosynthetic\_gene\_cluster | 31 | 21.4759169244 | 231 | 1.2e-59 |
| ABS74181.1 | BmyA | BGC0001090 | Bacillomycin\_biosynthetic\_gene\_cluster | 31 | 24.4365885992 | 230 | 2e-59 |
| AAN85522.1 | hybrid\_nonribosomal\_peptide\_synthetase\_/\_polyketide\_synthase | BGC0001101 | Leinamycin\_biosynthetic\_gene\_cluster | 30 | 24.701723376 | 229 | 3.4e-59 |
| AAM12925.2 | MmpE | BGC0000182 | Mupirocin\_biosynthetic\_gene\_cluster | 31 | 23.5969951392 | 229 | 4.4e-59 |
| BAP05590.1 | CalB | BGC0000967 | Calyculin\_biosynthetic\_gene\_cluster | 32 | 22.182942996 | 228 | 5.7e-59 |
| BAA89382.2 |  | BGC0000864 | Eicosapentaenoic\_acid-like\_compound\_biosynthetic\_gene\_cluster | 25 | 39.4608926204 | 228 | 9.8e-59 |
| ACM79805.1 | ZmaA | BGC0001059 | Zwittermycin\_A\_biosynthetic\_gene\_cluster | 29 | 29.4741493593 | 225 | 6.3e-58 |
| ABS90478.1 | PKS | BGC0001106 | Oxazolomycin\_biosynthetic\_gene\_cluster | 28 | 25.8506407424 | 218 | 7.7e-56 |
| AEH59110.1 | polyketide\_synthase\_of\_type\_I | BGC0000385 | Lysobactin\_biosynthetic\_gene\_cluster | 30 | 22.8015908087 | 217 | 2.2e-55 |
| AGJ76601.1 | HglE | BGC0000869 | Heterocyst\_glycolipids\_biosynthetic\_gene\_cluster | 27 | 32.7441449403 | 212 | 4.2e-54 |
| CAL69888.1 | RhiA\_protein | BGC0001112 | Rhizoxins\_biosynthetic\_gene\_cluster | 29 | 22.8457799381 | 212 | 5.5e-54 |
| AFR69332.1 | polyketide\_synthase\_SpiB | BGC0001045 | Spiruchostatin\_biosynthetic\_gene\_cluster | 31 | 22.8015908087 | 212 | 5.5e-54 |
| BAP05596.1 | CalH | BGC0000967 | Calyculin\_biosynthetic\_gene\_cluster | 29 | 24.5249668582 | 210 | 1.6e-53 |
| AAM12934.1 | MmpF | BGC0000182 | Mupirocin\_biosynthetic\_gene\_cluster | 28 | 29.076447194 | 210 | 2.7e-53 |
| ABC34154.1 | thiotemplate\_mechanism\_natural\_product\_synthetase | BGC0000961 | Bactobolin\_biosynthetic\_gene\_cluster | 29 | 23.5528060097 | 209 | 4.7e-53 |
| AAV97877.1 | OnnI | BGC0001105 | Onnamide\_biosynthetic\_gene\_cluster | 29 | 24.701723376 | 208 | 8e-53 |
| CAJ76289.1 | putative\_hybrid\_non-ribosomal\_peptide-polyketide\_synthetase | BGC0000972 | Colibactin\_biosynthetic\_gene\_cluster | 29 | 23.6411842687 | 207 | 2.3e-52 |
| CBF69451.1 | polyketide\_synthase,\_putative\_(JCVI) | BGC0000037 | Cichorine\_biosynthetic\_gene\_cluster | 24 | 49.4034467521 | 207 | 2.3e-52 |
| CAD15512.1 | probable\_polyketide\_synthase\_protein | BGC0001014 | Micacocidin\_biosynthetic\_gene\_cluster | 29 | 24.5249668582 | 205 | 6.8e-52 |
| AHD05620.1 | putative\_polyketide\_synthase\_subunit | BGC0001033 | Paenilamicin\_biosynthetic\_gene\_cluster | 26 | 29.5183384887 | 204 | 1.2e-51 |
| BAV19380.1 | NRPS-like\_enzyme | BGC0001390 | LL-Z1272beta\_biosynthetic\_gene\_cluster | 28 | 23.110914715 | 198 | 8.3e-50 |
| CAJ76297.1 | putative\_polyketide\_synthase | BGC0000972 | Colibactin\_biosynthetic\_gene\_cluster | 27 | 29.3857711003 | 197 | 1.4e-49 |
| CAJ76285.1 | putative\_polyketide\_synthase | BGC0000972 | Colibactin\_biosynthetic\_gene\_cluster | 27 | 29.4741493593 | 197 | 1.8e-49 |
| BAB69199.1 | modular\_polyketide\_synthase | BGC0000117 | Oligomycin\_biosynthetic\_gene\_cluster | 26 | 25.718073354 | 180 | 2.3e-44 |
| BAF50720.1 | hybrid\_non\_ribosomal\_peptide\_synthetase-polyketide\_synthase | BGC0001116 | Virginiamycin\_biosynthetic\_gene\_cluster | 26 | 25.3645603182 | 179 | 5.2e-44 |
| CAE46850.1 | Type\_I\_modular\_polyketide\_synthase | BGC0000103 | Mycolactone\_biosynthetic\_gene\_cluster | 30 | 20.3269995581 | 176 | 2.6e-43 |
| AAM94794.1 | CalE8 | BGC0000033 | Calicheamicin\_biosynthetic\_gene\_cluster | 25 | 38.7980556783 | 176 | 3.4e-43 |
| AAQ17110.2 | enediyne\_polyketide\_synthase | BGC0001008 | Maduropeptin\_biosynthetic\_gene\_cluster | 25 | 33.6279275298 | 173 | 2.2e-42 |
| AGN74895.1 | nonribosomal\_peptide\_synthetase/polyketide\_synthase\_hybrid\_protein | BGC0000459 | Griseoviridin\_/\_viridogrisein\_biosynthetic\_gene\_cluster | 28 | 20.8130799823 | 166 | 4.6e-40 |
| ACY06292.1 | modular\_polyketide\_synthase | BGC0001042 | Sanglifehrin\_A\_biosynthetic\_gene\_cluster | 31 | 20.4153778171 | 160 | 3.3e-38 |
| ABP73647.1 | SalC | BGC0000145 | Salinosporamide\_A\_biosynthetic\_gene\_cluster | 26 | 22.3155103844 | 149 | 3.4e-35 |
| ABP53496.1 | Erythronolide\_synthase | BGC0001041 | Salinosporamide\_biosynthetic\_gene\_cluster | 26 | 22.3155103844 | 149 | 3.4e-35 |
| ADC45535.1 | modular\_polyketide\_synthase | BGC0000093 | Meilingmycin\_biosynthetic\_gene\_cluster | 25 | 20.9898365002 | 137 | 1.7e-31 |
| AGJ76603.1 | HglC | BGC0000869 | Heterocyst\_glycolipids\_biosynthetic\_gene\_cluster | 25 | 23.2876712329 | 134 | 1.9e-30 |
| AAG31130.1 | MxcG | BGC0001345 | Myxochelin\_biosynthetic\_gene\_cluster | 26 | 23.4644277508 | 130 | 2.1e-29 |
| AAL01060.1 | omega-3\_polyunsaturated\_fatty\_acid\_synthase\_PfaA | BGC0000865 | Eicoseicosapentaenoic\_acid\_biosynthetic\_gene\_cluster | 23 | 20.0618647813 | 84 | 1.3e-15 |
| ACI12948.1 | PfaA | BGC0000861 | Eicosapentaenoic\_acid\_biosynthetic\_gene\_cluster | 22 | 20.7247017234 | 84 | 1.3e-15 |
| AFP73394.1 | FusA | BGC0001268 | Fusarin\_biosynthetic\_gene\_cluster | 21 | 23.0667255855 | 55 | 1.1e-06 |
